# Proteome-Wide Identification of RNA-Dependent Proteins in Lung Cancer Cells

**DOI:** 10.3390/cancers14246109

**Published:** 2022-12-12

**Authors:** Varshni Rajagopal, Astrid-Solveig Loubal, Niklas Engel, Elsa Wassmer, Jeanette Seiler, Oliver Schilling, Maiwen Caudron-Herger, Sven Diederichs

**Affiliations:** 1 Division of RNA Biology & Cancer, German Cancer Research Center (DKFZ), 69120 Heidelberg, Germany; 2 Division of Cancer Research, Department of Thoracic Surgery, Medical Center—University of Freiburg, Faculty of Medicine, University of Freiburg, German Cancer Consortium (DKTK)—Partner Site Freiburg, 79106 Freiburg, Germany; 3 Institute for Surgical Pathology, Medical Center—University of Freiburg, Faculty of Medicine, University of Freiburg, German Cancer Consortium (DKTK)—Partner Site Freiburg, BIOSS Centre for Biological Signaling Studies, 79106 Freiburg, Germany

**Keywords:** RNA, RNA-dependent proteins, RNA-binding proteins, R-DeeP, Sucrose density gradients

## Abstract

**Simple Summary:**

In this study, we identify RNA-dependent proteins in A549 lung cancer cells in a proteome-wide unbiased screen to elucidate the ribonucleoprotein complexes present in cancer cells. This dataset will help to understand and further dissect the role of RNA and RNA-dependent proteins in lung cancer. It is presented in the user-friendly R-DeeP 2.0 database which provides detailed information about RNA-dependent proteins and their properties in A549 along with data from HeLa cells to the scientific community.

**Abstract:**

Following the concept of RNA dependence and exploiting its application in the R-DeeP screening approach, we have identified RNA-dependent proteins in A549 lung adenocarcinoma cells. RNA-dependent proteins are defined as proteins whose interactome depends on RNA and thus entails RNA-binding proteins (RBPs) as well as proteins in ribonucleoprotein complexes (RNPs) without direct RNA interaction. With this proteome-wide technique based on sucrose density gradient ultracentrifugation and fractionation followed by quantitative mass spectrometry and bioinformatic analysis, we have identified 1189 RNA-dependent proteins including 170 proteins which had never been linked to RNA before. R-DeeP provides quantitative information on the fraction of a protein being RNA-dependent as well as it allows the reconstruction of protein complexes based on co-segregation. The RNA dependence of three newly identified RNA-dependent proteins, DOCK5, ELMO2, also known as CED12A, and ABRAXAS1, also known as CCDC98, was validated using western blot analysis, and the direct RNA interaction was verified by iCLIP2 for the migration-related protein DOCK5 and the mitosis-related protein ABRAXAS1. The R-DeeP 2.0 database provides proteome-wide and cell line-specific information from A549 and HeLa S3 cells on proteins and their RNA dependence to contribute to understanding the functional role of RNA and RNA-binding proteins in cancer cells.

## 1. Introduction

In recent years, the non-coding part of the genome has gained significant attention due to its crucial role in normal developmental processes and diseases [1]. Although most of the genome is actively transcribed, only 2% of the entire genome codes for proteins. RNAs that do not code for proteins are known as non-coding RNAs (ncRNA). Depending on their size, they are classified as small non-coding RNAs, which include microRNA (miRNA), small interfering RNA (siRNA), etc., that are shorter than 200 nucleotides (nt) versus the heterogeneous group of long non-coding RNAs (lncRNA) [2].

MiRNAs are short ncRNAs generated by the RNases Drosha and Dicer, which function as regulators of gene expression in complex with an Argonaute protein [3]. Several studies have reported the essential role of miRNAs in various cellular processes such as cell proliferation, growth, apoptosis and many human diseases, and most importantly in cancer [3,4,5]. Initially, lncRNAs were misunderstood as transcriptional noise resulting from the limited fidelity of RNA polymerase [6]. Later, lncRNAs were discovered to be important for various cellular processes such as chromosome dynamics, telomere biology, structural organization, and transcriptional and post-transcriptional processing [2,6]. In addition, numerous studies linked lncRNAs to human diseases due to their aberrant expression and function and ability to regulate various protein-coding genes [7,8]. LncRNAs have emerged as important players in different cancer types including lung cancer [2,9].

Lung cancer is the most common cause of cancer-related death contributing 18% to the cancer-related deaths worldwide in 2020 [10]. More than 80% of lung cancer is classified as non-small cell lung cancer (NSCLC) with a poor 5-year survival rate [11]. In lung cancer, dysregulated lncRNAs play a pivotal role in carcinogenesis and progression by regulating genes involved in signaling networks at transcriptional, post-transcriptional and translational levels, thereby modifying malignancy and treatment responses [12]. For example, a high expression of MALAT1 is associated with metastasis and poor prognosis in lung adenocarcinoma [13,14]. The downregulation of MALAT1 inhibits autophagy, henceforth inhibiting NSCLC progression [14]. The lncRNA LINC00473 interacts with the non-POU domain-containing octamer-binding protein and is upregulated in NSCLC contributing to poor prognosis [15]. The knockdown of LINC00473 promotes radiosensitivity in NSCLC [16]. Several other lncRNAs such as PVT1, LINC00673, lincNMR or VELUCT play a critical role in lung cancer progression depending on their interacting partners and expression levels [17,18,19,20,21]. Similarly, several studies have emphasized the significant role of miRNA in lung cancer [5,22,23,24,25,26]. One such study showed that a reduced expression of *let-7* associates with the reduced post-operative survival of lung cancer patients [23]. Hence, understanding and characterizing the molecular players at the RNA and the protein level and their alterations are critical for the early diagnosis and effective treatment of lung cancer [27].

Interactions between proteins and RNAs forming ribonucleoprotein complexes or RNPs are important for basic biological processes such as translation, splicing and localization [28]. The mammalian genome encodes between 600 and 6000 RNA-binding proteins (RBPs) depending on the approach used for identification [28,29]. The composition of RNPs is dynamic and dependent on the maturation state of the RNA and other cellular and environmental stimuli [30]. The RNP composition can vary from the interaction of a single protein with a single RNA to the formation of complex assemblies of multiple proteins and multiple RNAs [31]. RBPs have been implicated in various cellular processes in health and disease [32]. They are deregulated in various human diseases including cancer, which results in modulated cell phenotypes and pathological conditions. They are key players of cancer progression and development [33,34]. For example, altered RBPs can lead to differential splicing events in cancer cells, thus affecting various cancer hallmarks [35]. Hence, RBPs are considered to be therapeutic targets for cancer treatment [35].

Numerous studies have shed light on the central role of various RNAs and their interacting proteins that are vital for their biogenesis and functions [36,37,38]. Therefore, it is important to have a comprehensive understanding of the RBP repertoire in human cancer cells. Multiple proteome-wide methods have been applied to screen for RBPs which are mostly based on pull-down assays of polyadenylated (poly(A)+) RNA-interacting proteins [7,28,39,40]. In addition to canonical RNA-binding domains, such studies have reported an enrichment of intrinsically disordered regions. Many studies were restricted to poly(A)+ RNAs highlighting the importance of alternative strategies to identify the full repertoire of human RBPs [41]. Orthogonal methods have been developed to detect RBPs interacting with non-poly(A)+ RNA that depend on their physicochemical properties, modified nucleotides or protease digestion to map the RNA-binding regions [42,43,44]. Strategies using RNase digestion or phenol-chloroform extraction were developed such as DIF-FRAC that represents a complementary dataset to understand functions and the pathological relevance of RBPs [45]. Currently, several databases are available that include RBPs based on experimental data, literature search and computational analysis [29,46,47,48]. When comparing proteome-wide approaches for RBP identification from 43 human datasets, notably 2434 proteins were found only in one of the studies and thus do not overlap with other studies, while 3666 were common to at least two studies [29]. To develop a complementary method of RBP identification and also gain quantitative information about the RNA–protein interaction, we recently developed the concept of RNA dependence, defining a protein as RNA-dependent if its interactome depends on RNA which we directly translated into the R-DeeP approach that identifies RNA-dependent proteins proteome-wide and quantitatively and is independent of any potentially biased affinity- or physicochemical property-based purifications [30,49]. Briefly, proteins and protein complexes that were dependent on RNA were identified using sucrose density gradient ultracentrifugation, fractionation and a comparison of the apparent molecular weight of the protein complexes in the presence or absence of RNA. Using this method, more than 500 novel RNA-dependent proteins had been found in HeLa cells [30]. Here, we screened for RNA-dependent proteins proteome-wide in a non-small cell lung cancer model using the R-DeeP approach to expand our knowledge of the human RBPome. We identified and validated novel RNA-dependent proteins, corroborating the analysis of RNA–protein complexes in lung cancer. We made this resource also freely available on a versatile web-based platform, R-DeeP2.0 available at https://R-DeeP2.dkfz.de/ (accessed on 11 January 2022).

## 2. Material and Methods 

### 2.1. Cell Culture

A549 cells (ATCC^®^ CCL-185^TM^) were grown in RPMI 1640 medium (Gibco, ThermoFisher Scientific, Waltham, MA, USA) supplemented with 10% FBS (ThermoFisher Scientific, Waltham, MA, USA). Cells were cultured in a humidified incubator at 37 °C with 5% CO_2_.

### 2.2. Cell Harvest

For sucrose density gradient: Adherent A549 cells were harvested at a confluency of 90% from two 15 cm plates per gradient in the following way: cells were washed first with 10 mL pre-warmed PBS and then detached from the plate in 10 mL of cold (4 °C) PBS by using a cell scraper and centrifuged at 800× *g* for 5 min at 4 °C. The supernatant was discarded before lysis.

For frozen cell pellets in IT-buffer (25 mM Tris-HCl pH 7.5, 137 mM NaCl, 5 mM KCl, 0.5 mM MgCl_2_, 0.7 mM CaCl_2_, 0.3 mM Na_2_HPO_4_, protease inhibitors) and 50% glycerol, cells were transferred into a 50 mL centrifugation tube containing 10 mL of ice-cold IT buffer. The supernatant was removed, and cells were washed twice with 10 mL ice-cold IT buffer by centrifugation at 800× *g* for 5 min at 4 °C. The buffer was completely removed before lysis.

For iCLIP2, 6 million A549 cells were seeded in a 15 cm plate (10 dishes/CLIP) with 20 mL RPMI 1640 medium (Gibco, ThermoFisher Scientific, Waltham, MA, USA) supplemented with 10% FBS (ThermoFisher Scientific, Waltham, MA, USA). After 24 h, the medium was discarded, and the cells were washed once with 10 mL cold PBS (4 °C). Then, 15 mL cold PBS was added to the plate. The cells were UV-crosslinked at 254 nm on ice at 200 mJ/cm^2^ energy. The cells were then detached from the plate in 10 mL of cold PBS by using a cell scraper and centrifuged at 800× *g* for 5 min at 4 °C. The supernatant was discarded, and the pellet was snap-frozen in liquid nitrogen and stored at −80 °C until cell lysis.

### 2.3. Lysate Preparation

Cells were lysed by adding 100 µL of lysis buffer (25 mM Tris-HCl pH 7.4, 150 mM KCl, 0.5 % (*v/v*) NP-40, 2 mM EDTA, 1 mM NaF, 0.5 mM DTT, protease inhibitor) per 2 × 10^7^ cells, incubated on ice for 30 min with brief vortexing every 5 min. After three freezing–thawing cycles, cells were homogenized with a 0.4 mm needle. The cell debris was removed by centrifugation (16,000× *g*, 10 min, 4 °C).

### 2.4. RNase Treatment

First, 2 mg of cleared lysate was either treated with an RNase cocktail (per 100 µL cell extract 10 µg RNase A, 10 U RNase I, 1000 U RNase T1, 5 U RNase H and 1 U RNase III) or a solvent control for 1 h at 4 °C to digest the RNA.

### 2.5. Ultracentrifugation and Fractionation

The digested samples were then loaded on top of previously prepared sucrose gradients (gradient 5% to 50% sucrose in 5% steps and 1 mL volume per step) and separated by ultracentrifugation (Sorvall WX+ Ultracentrifuge from Thermo Scientific with a SW41 Ti-Rotor) overnight (18 h, 110,000–115,000× *g*, 4 °C). The gradient was fractionated in 395 µL aliquots (25 fractions per gradient). The samples were snap-frozen in liquid nitrogen and stored at −80 °C for later use for Western blot validation or mass spectrometry analysis. For a detailed protocol, refer to [30].

### 2.6. SDS-PAGE and Western Blot Analysis

Sodium dodecyl sulfate–polyacrylamide gel electrophoresis (SDS-PAGE) was performed to separate proteins according to their molecular weight. The samples containing 1× SDS sample buffer (30% (*v/v*) glycerol, 12% (*w/v*) SDS, 3.6 M DTT, 0.012% (*w/v*) bromophenol blue, and 500 mM Tris-HCl (pH 6.8)) were boiled at 95 °C for 5 min and shortly centrifuged afterwards. Then, 15 µL of the samples was loaded onto a 4–20% Criterion™ TGX Stain-Free™ Protein Gel with 26 wells (Bio-Rad, cat no. 5678095, Hercules, CA, USA) and run at 90 V in the electrophoresis chamber containing 1x SDS Running buffer (25 mM Tris base, 192 mM glycine, 0.1% (*w/v*) SDS). Precision Plus Protein™ Kaleidoscope™ Standards were used as protein size markers.

The proteins were transferred onto a nitrocellulose membrane using the Transblot Turbo wet transfer system using 1× of 5× Trans-Blot Turbo Transfer Buffer (Bio-Rad, cat no. 10026938, Hercules, CA, USA) containing 20% ethanol (2.5 A, 25 V, 10 min, Midi gel). The membrane was blocked with 5% milk in Tris-buffered saline (24.7 mM Tris-HCl (pH 7.4), 137 mM NaCl, 2.7 mM KCl) containing 0.05% Tween-20 (TBST) for 1 h at RT. Furthermore, the membrane was incubated at 4 °C overnight with the respective antibodies hnRNPU (Santa Cruz, 1:1000 dilution in 5% milk-TBST, cat no. sc-32315, Dallas, TX, USA), Asparagine Synthase (Santa Cruz, 1:1000 dilution in 5% milk-TBST, sc-365809, Dallas, TX, USA), ELMO2 (Abcam, 1:1000, ab181234, Cambridge, UK), DOCK5 (Bethyl laboratories, 1:500 in 5% milk-TBST, A304-988A-M, Montgomery, TX, USA), CCDC98/ABRAXAS1 (Abcam, 1:1000 dilution in 5% milk-TBST, ab139191-100, Cambridge, UK) overnight at 4 °C on a shaker. The next day, the membrane was washed three times for 5 min with TBST and incubated with the appropriate secondary HRP-conjugated antibody (Goat anti-mouse IgG-HRP (Dianova, 115-035-003, Hamburg, Germany) or Goat anti-rabbit IgG-HRP (Dianova, 111-035-144, Hamburg, Germany) diluted 1:5000 in blocking solution for 1 h at RT. The membrane was then washed three times for 5 min with TBST at room temperature. To visualize the protein bands, the membrane was incubated with the ECL reagent (Super Signal West PICO chemiluminescence substrate, ThermoFisher Scientific, 34580, Waltham, MA, USA) for 5 min and developed using an INTAS ECL Chemocam imager. 

### 2.7. Quantitative Analysis of the Western Blot Images

Quantitative analysis of Western blot images was performed using the software ImageJ. The sum of the Western blot signal over the 25 fractions was normalized to 100 in order to be compared to the mass spectrometry analysis.

### 2.8. Individual Nucleotide Resolution and UV Crosslinked Immunoprecipitation (iCLIP2)

Cell lysis: Eight UV crosslinked and two non-crosslinked frozen cell pellets were thawed per experiment and resuspended and lysed in 1 mL lysis buffer per pellet containing (50 mM Tris-HCl pH 7.4, 100 mM NaCl, 1% Igepal (CA-630), 0.1% SDS, 0.5% Sodium deoxycholate, protease inhibitor cocktail). The protein amount was measured using a BCA assay. The lysate was distributed into 1.5 mL low-bind tubes (1 mL/tube—later using 2 mL/IP). The lysates were treated with different RNase I concentrations (RNase I, Ambion AM2295, Austin, Texas, USA, pre-diluted in lysis buffer) ranging from 1:2.5 to 1:500 dilutions for DOCK5 and ABRAXAS1 iCLIP2 for 3 min at 37 °C at 1100 rpm on a thermomixer (ThermoMixer C, Eppendorf, EP5384000012, Hamburg, Germany). After RNase I treatment, the samples were incubated on ice for 3 min and centrifuged at 17,000× *g* for 20 min at 4 °C. The supernatant was collected and filtered through a proteus clarification mini spin column (SERVA Electrophoresis GmbH, 42225.01, Heidelberg, Germany) by centrifuging at 16,000× *g* for 2 min at 4 °C. The filtered lysates were then transferred to fresh 2 mL low-bind tubes (1 tube/condition) and kept on ice.

Beads/antibody preparation: Meanwhile, 500 µL (100 µL/IP) Dynabeads Protein A (ThermoFisher Scientific, 10002D, Waltham, MA, USA) was washed three times with 1 mL lysis buffer, split into two tubes for the IgG control (100 µL) or the protein of interest DOCK5/ABRAXAS1 (400 µL), incubated with the antibody for DOCK5 iCLIP2 (IgG/DOCK5 12 µg/IP) or for ABRAXAS1 iCLIP2 (IgG/ABRAXAS1: 1 µg/IP) for 1 h at RT at a rotation of 10 rpm. IgG: Normal Rabbit IgG, (Millipore 12-370, Burlington, MA, USA); DOCK5: Anti-DOCK5 (Biomol A3049887, Hamburg, Germany); ABRAXAS1/CCDC98: Anti-CCDC98 (Abcam EPR6310(2) 139191, Cambridge, UK). After 1 h, bead–antibody complexes were captured on a magnetic rack and washed once with 1 mL high salt wash buffer (50 mM Tris-HCl pH 7.4, 1.5 M NaCl, 1 mM EDTA pH 8.0, 1% Igepal CA-630, 0.1% SDS, 0.5% Sodium deoxycholate) and twice with 1 mL lysis buffer. The beads were then resuspended in 100 µL lysis buffer per IgG or 400 µL lysis buffer per DOCK5 or ABRAXAS1 pulldown.

Immunoprecipitation: The resuspended beads were then added to the respective tubes containing cleared lysate (100 µL/IP) and incubated for 2 h on a rotation wheel (10 rpm) at 4 °C for immunoprecipitation. After 2 h, the complex was captured on a magnetic rack. The flow-through was removed, and the beads were washed twice with high salt wash buffer with rotation at 10 rpm at 4 °C for 1 min and then washed twice with 1 mL PNK wash buffer (20 mM Tris-HCl pH 7.4, 10 mM MgCl_2_, 0.2% Tween-20). During the last wash, the beads were transferred to fresh 1.5 mL low-bind tubes and stored at 4 °C. On the following day, 100 µL of beads was transferred to a fresh tube for Western blot and 900 µL was used for labeling the RNA. For Western blot, beads were captured, the supernatant was removed, and the protein complexes were eluted using 1× LDS buffer containing 50 mM DTT at 70 °C for 10 min. The eluate was collected and stored at −20 °C to check for immunoprecipitation efficiency using Western blot analysis. 

Radioactive labeling of RNA: The remaining 900 µL of the resuspended beads was used to label and check for RNA binding. For radioactive labeling of the bound RNA, PNK mix was prepared using 11.85 µL water, 0.75 µL T4 PNK (NEB, M0201S, Ipswich, MA, USA), 1.5 µL 10× PNK buffer (NEB, B0201S, Ipswich, MA, USA), 0.9 µL [γ-^32^P]ATP (EasyTides^®^ [gamma-32P]ATP, 250 µCi, PerkinElmer, NEG502A250UC, Waltham, MA, USA) per sample. Beads were captured on ice, the supernatant was removed and the beads were resuspended in 15 µL PNK mix and incubated on a thermomixer at 37 °C for 5 min at 1100 rpm for labeling the RNA. Furthermore, the samples were washed twice with 1 mL PNK wash buffer and eluted in 25 µL in 1× LDS buffer containing 50 mM DTT on a thermomixer at 70 °C for 10 min at 1100 rpm [50].

For SDS-PAGE, the samples were loaded onto a 7.5% Mini-PROTEAN^®^ TGX™ Precast Protein Gel (10-well, 50 µL, BioRad #4561024, Hercules, CA, USA) for DOCK5 and a 10% Mini-PROTEAN^®^ TGX™ Precast Protein Gel (10-well, 50 µL, BioRad #4561034, Hercules, CA, USA) for ABRAXAS1, run at 120 V in a vertical electrophoresis chamber filled with 1× SDS running buffer (25 mM Tris base, 192 mM glycine, 0.1% (*w/v*) SDS). The proteins were transferred from the gel onto a nitrocellulose membrane (Amersham Protran 0.45 µm Nitrocellulose, Fisher Scientific 10600002, Schwerte, Germany) using a wet transfer system with wet transfer buffer (25 mM Tris base, 192 mM glycine) containing 20% methanol for 1.5 h at 120 V in an ice bath.

After the transfer, the membrane was washed once in nuclease-free water, covered with plastic wrap and exposed to a phosphor imager screen. After an appropriate amount of time, the screen was imaged on a phosphor imager at 200 µm, high speed and intensity 3. 

### 2.9. Sample Preparation for Mass Spectrometry

For mass spectrometry, 200 µL of each fraction was used for TCA precipitation. One volume of 100 mM HEPES pH7.5/2 mM TCEP/4 mM Iodoacetamide was added, and the mixture was incubated for 30 min at room temperature. Next, 100 µL 100% TCA was added, the sample was vortexed and incubated for 15 min on ice followed by centrifugation (15 min, 16,000× *g*, 4 °C). The pellet was washed three times with 10% (*v/v*) TCA and three times with cold 100% acetone (in between centrifugation steps: 10 min, 4 °C, 16,000× *g*). The washed pellet was air-dried.

For trypsin digestion, the pellet was dissolved in 100 mL 50 mM HEPES/0.1% RapiGest with 30 min incubation in an ultrasonic bath with ice. Then, 1 µg trypsin (Life Technologies) was added to each sample and the digestion was completed overnight at 37 °C in an incubator.

The TMT-6-plex labels (ThermoFisher Scientific, Waltham, MA, USA) were dissolved in water-free DMSO (0.8 mg in 44 µL DMSO) and 5 µL label was used per sample. The samples were shortly vortexed and then incubated overnight at room temperature on a shaker. Afterwards, the differentially labeled samples from the same fraction were mixed (Fraction 1 from −/+ RNase in triplicates = six labels and so on).

Next, the samples were desalted using HyperSpin TipC-18 columns (ThermoFisher Scientific, Waltham, MA, USA). The column was equilibrated with 100 µL 100% acetonitrile and washed three times with 100 µL 0.1% TFA/8% acetonitrile (in between centrifugation steps always 3 min, 500× *g*, room temperature). After sample loading, the column was washed three times with 100 µL 10% acetonitrile, and then, the sample was eluted with 60 µL 65% acetonitrile. 

For mass spectrometry analysis, 2 µg peptides per sample was dried in a speed vac. The samples were further analyzed using the Liquid Chromatography-Mass Spectrometry technique. Afterwards, the data were analyzed in our R-DeeP-Pipeline. In brief, this includes the following steps: normalization between replicates, finding the fit parameters from average curves, perform Gaussian fit on each curve, quality control, assessment of p-values and evaluation of the shifts. For a detailed protocol on sample preparation, mass spectrometry analysis and data analysis, refer to our previous publication [30]. 

## 3. Results

### 3.1. Proteome-Wide Identification of RNA-Dependent Proteins in Lung Cancer Cells Using the R-DeeP Approach

**Figure 1:**
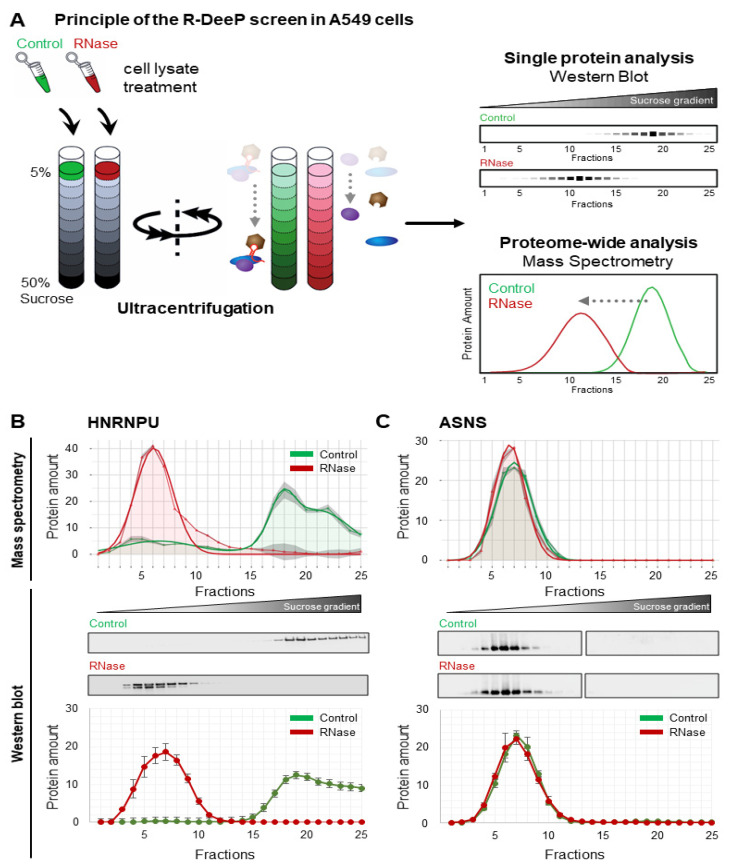
R-DeeP sucrose density gradient method and controls. (**A**) A549 lysates were left untreated (Control) or treated with RNase and loaded onto the sucrose gradients, which were then subjected to ultracentrifugation and fractionated into 25 fractions. The triplicates of the gradients were analyzed using mass spectrometry or Western blot. Adapted from [49]. (**B**) The top panel shows the mass spectrometry analysis of the protein HNRNPU depicting the distribution of protein amount across the 25 fractions in the control and RNase-treated gradients. Raw data showing the mean of three replicates is indicated by the curve with points and the smooth curve represents a Gaussian fit (Green: Control, Red: RNase-treated) that is part of the statistical analysis [30]. The overall protein amount was normalized to 100. The middle panel shows a representative example of the Western blot analysis of the protein HNRNPU and its distribution across 25 fractions in control and RNase-treated gradient samples. The bottom panel depicts the quantification of three replicates of the Western blot analysis (mean and SEM) for comparison to the mass spectrometry data. (**C**) Same as in 1B for the negative control protein ASNS. Original blots: see File S1.

In the R-DeeP screen, proteins are separated on a sucrose density gradient per ultracentrifugation in the presence or absence of RNase treatment followed by fractionation (Figure 1A). Each fraction from the density gradient can be further analyzed for individual proteins by Western blot or proteome-wide by mass spectrometry. Proteins are found in higher density fractions depending on the apparent molecular weight of their respective complex. RNA-dependent proteins, whose interactome depends on RNA, are identified by their apparent shift in the molecular weight in the absence of RNA: upon RNase treatment, the RNA-dependent proteins dissociate from their interactors and shift to lower density fractions (Figure 1A). This method aids in identifying and quantifying the RNA dependence of proteins without biases from other enrichment strategies. To validate the R-DeeP screen, two proteins were selected as controls and validated. HNRNPU (heterogeneous nuclear ribonucleoprotein U) served as a positive control of an RNA-dependent protein shifting in the gradient and ASNS (asparagine synthetase) served as a negative control with an unaltered interactome in the absence of RNA after RNase treatment (Figure 1B,C). All three replicates of the Western blots for validation were quantified and showed similar profiles comparable to the mass spectrometry dataset for these two proteins. Previous Crosslinking and Immunoprecipitation (CLIP) experiments followed by radioactive labeling of RNA confirmed the RNA-binding property for the positive control HNRNPU and the lack thereof for the negative control ASNS [30].

### 3.2. Analysis of the RNA-Dependent Shifts in the A549 R-DeeP Screen

**Figure 2:**
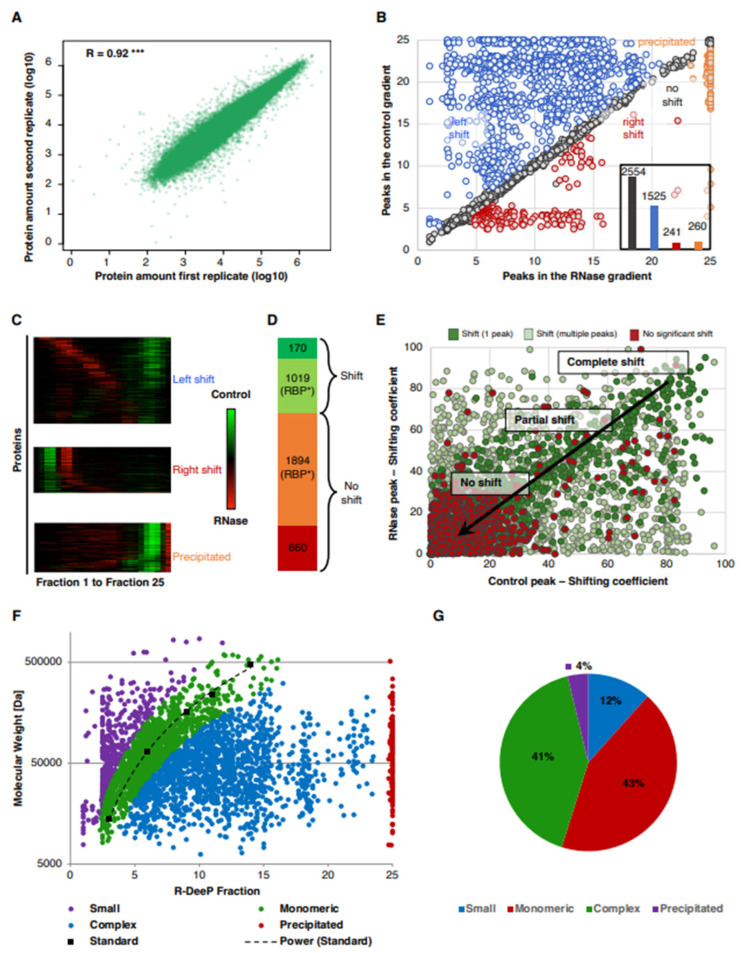
Proteome-wide R-DeeP screen in A549 cells. (**A**) The amount of each protein per fraction in different replicates after fraction-wise normalization is depicted documenting the reproducibility of the experiment. Pearson’s correlation coefficient R = 0.92, *** *p*-value < 0.001. (**B**) The graph shows the position of maxima in control and RNase-treated samples for each shift based on the mean of three replicates. The inset bar graph shows the number of proteins shifting left (blue) or right (red) or not (gray) or precipitating (orange). (**C**) A heatmap shows the significant shifts categorized for the direction of shift (left shift, right shift or precipitation). Green color represents protein enrichment in control fractions while red color represents protein enrichment in the RNase-treated fractions (**D**) The panel shows the number of shifting and non-shifting proteins detected in this R-DeeP screen grouped according to their identification as RBP candidates (RBP*) in 43 previous proteome-wide human studies as well as novel RBP candidates (*n* = 170). (**E**) The graph depicts the shifting co-efficient (protein amount at maxima × loss or gain by the shift) for each pair of control and RNase peaks. Red dots indicate proteins with no significant shifts, dark green depicts proteins with significant shifts between one control and one RNase-treated peaks and light green represents proteins with multiple peaks. The top right region of the graph represents proteins with a “complete shift” (almost the entire amount of the protein is shifting), bottom left indicates proteins with no shift and the candidates that lie in the middle are the proteins with a partial shift (a fraction of the protein is shifting, while another fraction does not show RNA dependence). (**F**) The graph shows the molecular weight and position of the maxima for the reference proteins in the gradient (black). The molecular weights of the reference proteins are RNase A (14 kDa), BSA (60 kDa), Aldolase (4 × 40 kDa = 160 kDa), Catalase (4 × 60 kDa = 240 kDa), and Ferritin (24 × 20 kDa = 480 kDa). The dashed black line shows the extrapolated relation between molecular weight of the protein and its position in the gradient. After RNase treatment, depending upon their position in the gradient, proteins were classified into smaller than monomeric size (purple), proteins at their monomeric size (green), proteins in a complex (blue) and precipitated proteins (red). (**G**) Pie chart indicating the percentage of proteins in each category smaller than monomeric size (purple), at monomeric size (green), in complex (blue) and precipitated (red).

We performed a proteome-wide R-DeeP screen in the lung adenocarcinoma cell line A549 using mass spectrometry analysis for three biological replicates with 25 control and RNase-treated fractions each, amounting to 150 samples. Mass spectrometry detected and quantified 3743 proteins across these samples. Our statistical pipeline to identify RNA-dependent proteins calculated Gaussian fitted distributions for each protein, and shifts were characterized according to different criteria: (a) amount of protein shifting in the presence or absence of RNA which was represented by the area under the curve; (b) the direction and distance of the shift; (c) position of the peak(s) of the curves in control and RNase-treated gradients reflecting the apparent molecular weight of the complex; (d) amplitude difference at each maximum between the control and RNase curves; and (e) the statistical significance of the difference at the amplitude maxima [30]. A shift was called, and the protein was categorized as RNA-dependent if: (a) the distance between the maxima in control and RNase-treated conditions was greater than one fraction; (b) the curves showed a significant difference at least at one of the maxima. The amount of each protein per fraction correlated well for all proteins between the three replicates after fraction-wise normalization indicating the reproducibility of the method (Figure 2A). The proteins were further grouped depending on the absence (no shift) or presence of a shift and for the latter according to the direction of the observed shift into left shifts (toward lower sucrose density fractions), right shifts (toward higher density fractions) and precipitated proteins (shifting to fraction >23). Out of the 3743 proteins detected in total and keeping in mind that proteins with multiple peaks could also show multiple shifts, we found 1525 left shifts (blue), 241 right shifts (red) and 260 precipitations after RNase treatment (orange), whereas no significant shift was observed for 2554 proteins (gray) (Figure 2B,C). In total, 1189 proteins were identified with at least one significant shift containing 170 novel RBP candidates, i.e., proteins which had not been found before in 43 proteome-wide human studies according to the comprehensive RBP2GO database (Figure 2D) [29]. In contrast, 1894 RBP candidates (RBP*) which had been identified in at least one previous proteome-wide RBP screen were detected in our screen but did not show RNA dependence (no significant shift). The average RBP2GO score for these non-shifting RBPs (11.65) was less than half the average score of the shifting RBPs (25.06). Furthermore, a shifting coefficient was calculated for each shift based on the quantification of the amount of protein present in a peak and its change upon RNase treatment. The proteins were then classified as RNA-independent (no shift), partially RNA-dependent (partial shift, i.e., only a fraction of the protein amount shifts) or completely RNA-dependent (complete shift) based on the shifting co-efficient reflecting the fraction of the total amount of a protein that shifted (Figure 2E). Since the position of a protein or complex in the gradient depends on its apparent molecular weight, the fraction information can be used to estimate the molecular weight calibrated relative to a reference of proteins of known molecular weight. Thereby, we determined the molecular weight of the proteins after RNase treatment finding that 43% of the proteins shifted roughly to its monomeric size, while 41% were still in a complex upon RNase treatment leading to a higher apparent molecular weight than expected from the monomeric protein size (Figure 2F,G). 

### 3.3. Properties of Shifting Proteins

**Figure 3:**
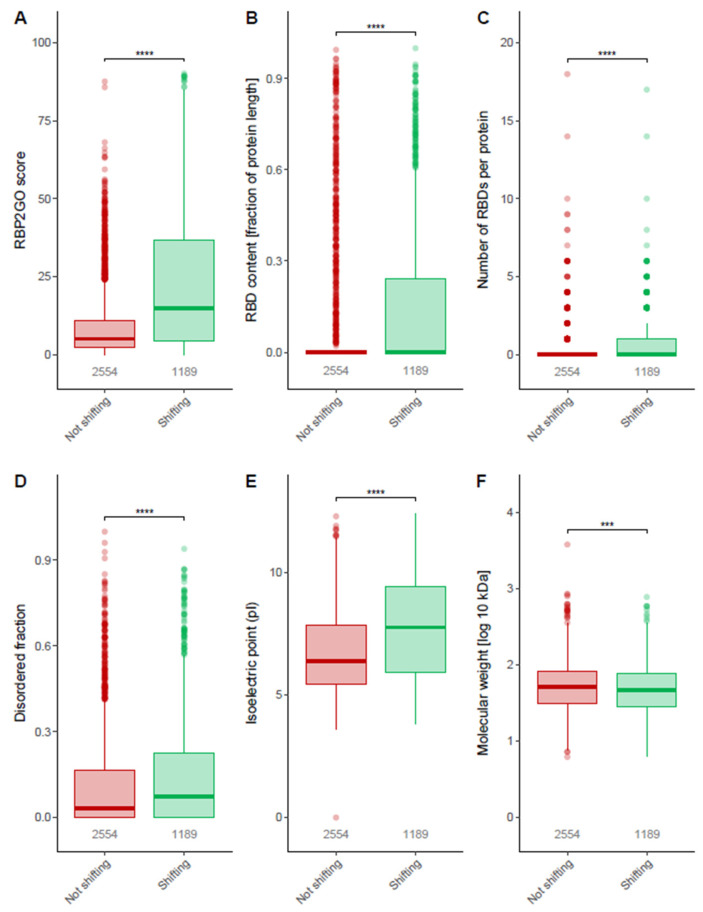
Properties of shifting proteins: (**A**) RBP2GO score of shifting vs. non-shifting proteins. (**B**) RNA binding domain (RBD) content fraction in shifting vs. non-shifting proteins. (**C**) Number of RBDs present in shifting vs. non-shifting proteins. (**D**) Intrinsically disordered region fraction in shifting vs. non-shifting proteins. (**E**) Isoelectric points (pI) of shifting vs. non-shifting proteins. (**F**) Molecular weight of shifting vs. non-shifting proteins. All: Data presented as boxplots with the bar indicating the median and the box indicating the lower and upper quartiles. The upper whisker extends from the hinge to the largest value not more than 1.5× interquartile range from the hinge and the lower whisker extends from the hinge to the smallest value at most 1.5× interquartile range of the hinge. The outliers are represented by dots. Wilcoxon test was used to calculate the *p*-value, (*** *p* < 0.001, **** *p ≤* 0.0001).

To further compare the groups of shifting and non-shifting proteins, we analyzed multiple properties related to RBPs. The RBP2GO score was—as expected—significantly and highly enriched in the shifting compared to the non-shifting proteins (Figure 3A). Furthermore, the shifting proteins possessed a significantly higher fraction of RBDs relative to their length compared to non-shifting proteins (Figure 3B) as well as a higher number of RBDs per protein (Figure 3C). Taken together, these three parameters indicate a strong and significant enrichment of RBPs in the group of shifting proteins. Disordered protein sequences termed “Intrinsically Disordered Regions” (IDRs) are sequences with low amino acid complexity that lack a defined structure. Additionally, IDRs have been linked to RNA binding properties of proteins supporting RNA–protein and protein–protein interactions [51,52]. Aptly, shifting proteins contain significantly higher disordered fractions relative to their length than non-shifting proteins (Figure 3D). When comparing the isoelectric points (pI), we found considerably higher pI values for shifting proteins than for non-shifting proteins (Figure 3E), which is in line with the capacity of positively charged amino acids to facilitate the binding of negatively charged RNA. Lastly, the molecular weight of the shifting proteins showed a minute but statistically significant difference between shifting and non-shifting proteins (Figure 3F).

For the validation of the RNA-dependent shifting and direct RNA-binding, we selected protein candidates based on the following criteria: proteins that had never been identified as RNA-binding before according to 43 human studies compiled in the RBP2GO database [29], the strength of the shift (distance, peak gain/loss) and the availability of suitable antibodies resulting in the selection of DOCK5, ELMO2 and ABRAXAS1 (CCDC98) for further analysis.

### 3.4. Validation of DOCK5 as RNA-Dependent Protein

**Figure 4:**
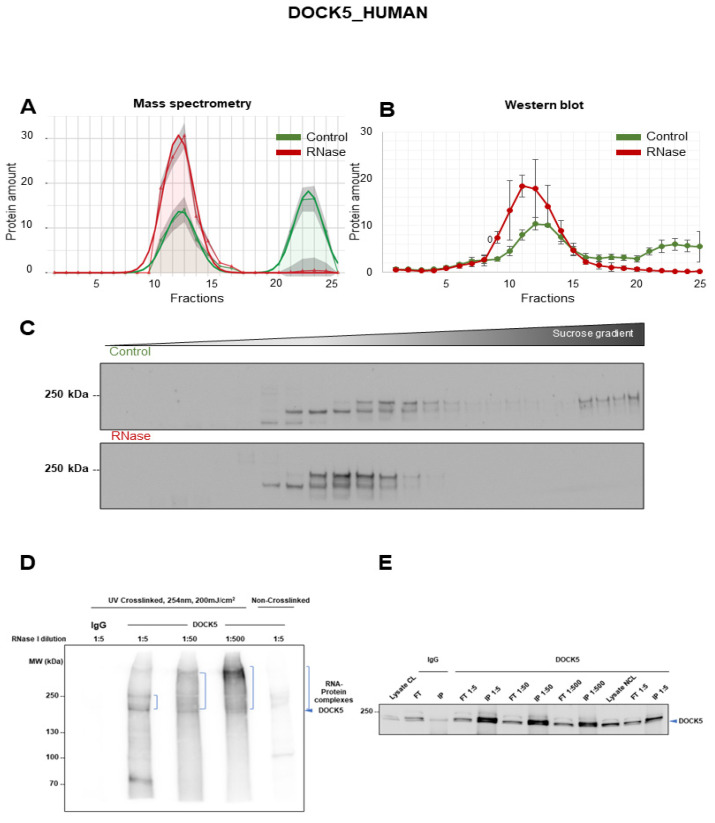
RNA dependence of DOCK5. (**A**) Graphical representation of protein amounts determined by mass spectrometry in all 25 fractions. The experiments were repeated in three replicates. Mean of the raw data (total protein amount normalized to 100) is indicated by the pointed curve with gray shadows representing the standard deviation while the smooth curve represents the Gaussian fit. (**B**) Graph representing the quantitative analysis of three Western blot replicates showing the mean and SEM across 25 fractions. The green line represents the control gradients, and the red line represents the RNase-treated gradients. The quantified overall protein amount was normalized to 100 to make it comparable to the mass spectrometry analysis. (**C**) Western blot image of DOCK5 (215 kDa) in A549 gradients treated −/+ RNase. (**D**) Autoradiography showing the direct RNA binding of DOCK5 by iCLIP2 indicated by shifting of the radioactive signal toward higher molecular weights with decreasing RNase I concentrations (*n* = 3). (**E**) Western blot confirming immunoprecipitation of DOCK5 (*n* = 3). Original blots: see File S1.

Dedicator of Cytokinesis 5 (DOCK5) belongs to the DOCK family of proteins that act as guanine nucleotide exchange factors (GEFs) for Rho GTPases such as Rac and Cdc42 [53]. DOCK5 proteins are evolutionarily well conserved and are classified into four subgroups based on their domain organization and sequence similarity. DOCK5 belongs to the DOCKA subgroup along with two other proteins, DOCK1 and DOCK2 [53]. DOCK5 is the closest homologue of DOCK1 and interacts with DOCK1 to mediate cell spreading and migration [53]. DOCK5 is the least studied member of the DOCK family of proteins, and it is known for its role in osteoclasts, cell migration, motility, invasion and murine embryonic development [54]. Together with DOCK1, DOCK5 is also involved in the generation of actin structures. DOCK5 mediates a Crk-p130Cas-DOCK5 signaling cascade to facilitate peripheral actin polymerization and membrane protrusion. Furthermore, the inhibition of DOCK5 reduces invasiveness and tumor burden in mice injected with MDA-MB-231 breast cancer cells [54]. Given the important role of DOCK5 in osteoclasts and various diseases such as cancer, we further validated the RNA dependence of DOCK5 discovered by mass spectrometry (Figure 4A) using Western blot quantification in A549 lung cancer cells (Figure 4B,C). The DOCK5 protein was present majorly in two different sets of fractions in the control gradient, fractions 9–14 and fractions 21–25. Upon RNase treatment, the protein is completely enriched in the earlier fractions (9–14) with no detectable signal in the later fractions (21–25). This pointed toward the dissociation of a DOCK5-containing complex in the absence of RNA making DOCK5 a (partially) RNA-dependent protein. In order to prove the direct RNA binding of DOCK5, the iCLIP2 protocol was used in A549 lysates [50]. In brief, after the DOCK5 IP, the interacting and co-purified RNA was labeled with [γ-^32^P] ATP. Autoradiography revealed the RNA binding to the immunoprecipitated protein. The immunoprecipitated RNA–protein complexes were exposed to different concentrations of RNase which gradually decreased the size of the bound RNA. Consequently, the size of the complex shifted downwards on the blot with increasing amounts of RNase due to the partial degradation and size reduction in the RNA verifying its identity as RNA. At the highest RNase concentration (1:5 dilution), this smear, the RNA signal, was most intense at the height of the DOCK5 protein, while a more dispersed smear toward higher molecular weights was observed at lower RNase concentrations (increasing dilutions: 1:50 and 1:500), proving that DOCK5 was an RNA binding protein (Figure 4D). As a control, the immunoprecipitation of DOCK5 was verified by Western blotting (Figure 4E).

### 3.5. Validation of ELMO2 as RNA-Dependent Protein

**Figure 5:**
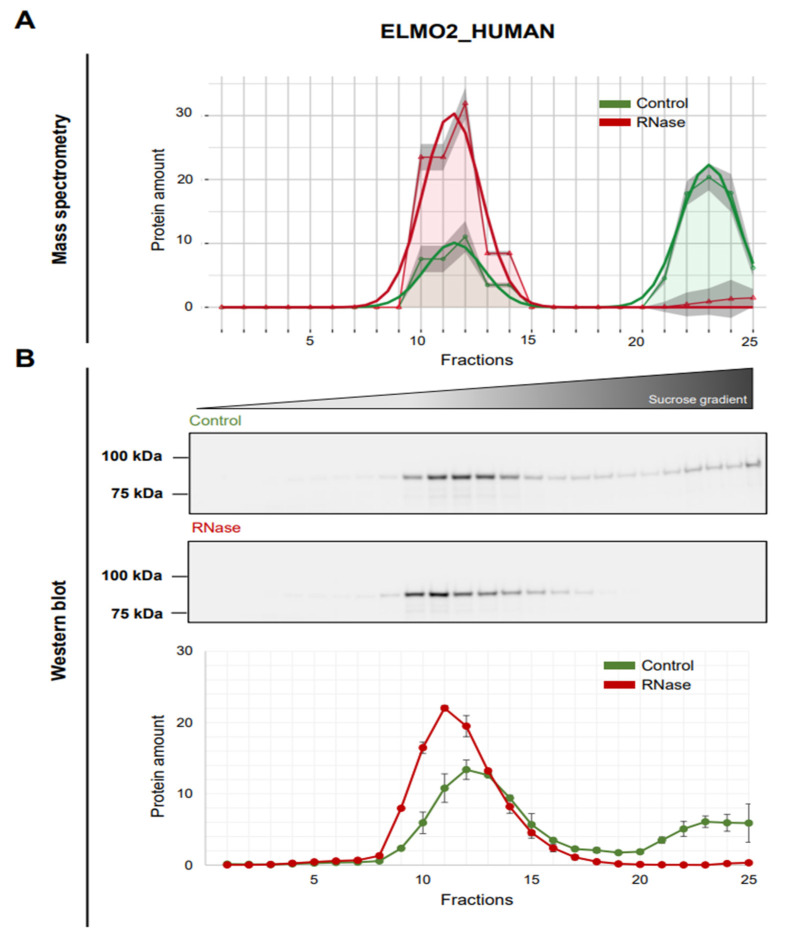
RNA dependence of ELMO2. (**A**) Mass spectrometry analysis of the ELMO2 protein in all 25 fractions as in Figure 4A. (**B**) Top panel: Western blot showing the protein amount of ELMO2 (83 kDa) distributed across the 25 fractions in control and RNase-treated samples. Bottom panel: Graph showing the quantitative analysis of the Western blots of all three replicates (*n* = 3) represented by mean and SEM. The protein amount was normalized to 100. Original blots: see File S1.

The “EnguLfment and cell MOtility” or ELMO family of proteins comprises three members, ELMO1, ELMO2 and ELMO3, that are evolutionarily conserved and are essential for engulfment and cell motility. They are one family of well-known interactors of the DOCK proteins [55,56]. Interaction with an ELMO protein is essential for the GEF activity of DOCK proteins to activate the Rac signaling pathway [56]. Rac-mediated actin cytoskeleton remodeling is known to be critical for cellular processes such as cell migration, myoblast fusion or phagocytosis [55]. Other than DOCK proteins, ELMO2, also known as CED12A, interacts with various other proteins such as Gαi2, Gβγ and Nck-1 that are involved in similar functions. Although ELMO2 has not been subject to extensive research in cancer, a recent study has reported that ELMO2 plays an important role in chemotaxis, invasion and migration mediated by CXCL-2 in pancreatic cancer [57]. In the mass spectrometry data of the R-DeeP screen in A549 cells, a substantial fraction of ELMO2 showed a shift from the fractions 21–25 to the fractions 9–14 upon RNA depletion (Figure 5A). These data were validated by Western blotting results correlating well with the mass spectrometry distribution (Figure 5B) verifying the partial RNA dependence of the ELMO2 protein. Notably, the patterns of DOCK5 and ELMO2 were highly similar regarding their distribution in the R-DeeP gradients which might also point toward their interaction. 

### 3.6. Validation of ABRAXAS1 as RNA-Dependent Protein

**Figure 6:**
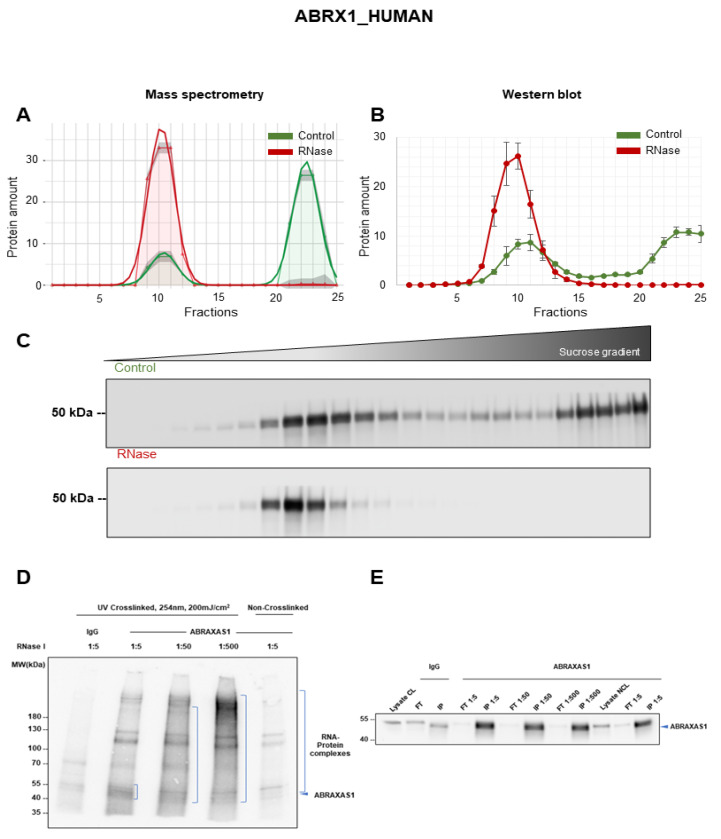
RNA dependence of ABRAXAS1. (**A**) Mass spectrometry analysis of the ABRAXAS1 (ABRX1_HUMAN) protein across the 25 fractions in control and RNase-treated samples (*n* = 3) as in Figure 4A. (**B**) Graph representing the quantitative analysis of ABRAXAS1 Western blots indicated by the mean of all three replicates and SEM. The protein amount was normalized to 100. (**C**) Western blot showing the protein distribution of ABRAXAS1 (47 kDa) across 25 fractions. (**D**) Autoradiography showing the direct RNA binding of ABRAXAS1 by iCLIP2 indicated by shifting of the radioactive signal toward higher molecular weights with decreasing RNase I concentrations (*n* = 3). (**E**) Western blot verifying immunoprecipitation of ABRAXAS1 (*n* = 3). Original blots see File S1.

The BRCA1-A complex subunit ABRAXAS1, also called ABRA1, CCDC98 or FAM175A, is one of the subunits of the BRCA1-A complex involved in the DNA damage repair pathway [58]. It is a coiled coil domain-containing protein that together with RAP80, BRE, and BRCC36 regulates the DNA damage checkpoint and DNA end resurrection in homologous recombination repair [58,59]. ABRAXAS1 depletion impedes the recruitment of BRCA1 to DNA damage sites and induces genomic instability [59]. In addition, ABRAXAS1 expression is reduced in various cancer entities and knockout mice exhibited decreased survival [59]. In the A549 R-DeeP mass spectrometry screen, ABRAXAS1 showed a major peak at fractions 20 to 25 in the control conditions with a small additional peak at fractions 8 to 13, while RNase treatment shifted the entire amount of the protein to this earlier peak (Figure 6A). Western blot analysis confirmed the RNA-dependent shift of ABRAXAS1 with a band at 47 kDa (Figure 6B,C). The direct RNA binding of ABRAXAS1 was validated using iCLIP2 as indicated by the gradual decrease in the height of the smear with increasing RNase I concentrations (Figure 6D). At the highest RNase I concentration (1:2.5 dilution), the signal is most intense at the size of the protein, while at lower RNase I concentrations (1:25 and 1:250 dilutions), the smear height is increasing. Western blotting confirmed the immunoprecipitation of ABRAXAS1 (Figure 6E).

### 3.7. R-DeeP 2.0 Database as a Versatile Resource to Analyze RNA-Dependent Proteins

**Figure 7:**
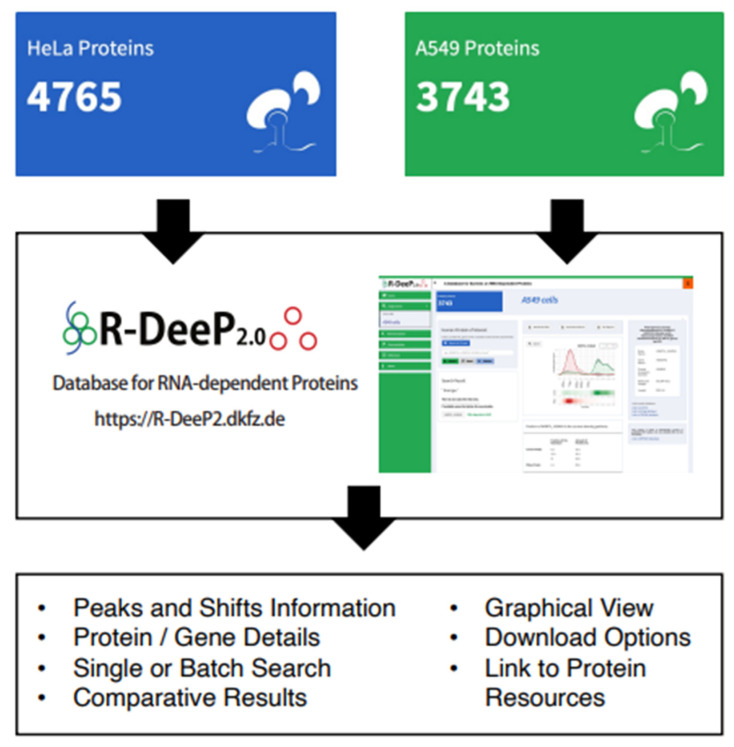
R-DeeP Database 2.0: The R-DeeP 2.0 database provides information on R-DeeP results on 4765 proteins in HeLa S3 cells and 3743 proteins in A549 cells at https://R-DeeP2.dkfz.de [22] (accessed on 11 January 2022). It offers multiple search and download options and returns information about each protein in terms of peaks and shifts in one cell line or both as well as graphical representations and other general information about the proteins of interest. It is linked to further external resources which give information about the interactors and protein complexes.

The R-DeeP 2.0 database (Figure 7) is available at https://R-DeeP2.dkfz.de (accessed on 11 January 2022) for public use. R-DeeP 2.0 contains the analyzed proteome-wide mass spectrometry data of proteins after sucrose density gradient fractionation in presence of RNA molecules and after RNase treatment in human HeLa S3 (previous dataset) [30] and A549 cells (new dataset). It provides various search options for the user and the summary of multiple RNA-binding protein resources with access to the corresponding publication webpages. The single search option allows searching the database for one protein of interest in either the HeLa S3 or the A549 cell line by entering the protein name or gene name or UniprotID. Using the advanced search option, the user can compare a protein directly between the two cell lines A549 and HeLa S3. In both cases, the complete R-DeeP 2.0 analysis results are available for download, which contain information such as graphical representation of the protein distribution in the gradients, results of the statistical quantitative analysis, the maxima, their position and the amount of protein for the control and RNase gradients, parameters of the shifts and further information about the protein. In addition, it indicates whether the protein of interest has already been listed as a potential RBP in previous studies and links directly to the RBP2GO database [29]. In addition, in the advanced search option, the protein data are automatically compared between HeLa S3 and A549 cells. For detailed instructions to use the database, kindly refer to the user guide which is available in the database under the documentation tab.

## 4. Discussion

Given the functional importance and complexity of RNA and RNA–protein interactions, several methods were developed to identify RBPs [28,39,42,60]. These methods were mostly based on affinity purification, UV-crosslinking, RNA pulldown or organic phase separation. Since each method has its own advantages and limitations as discussed previously [30], orthogonal strategies such as R-DeeP are needed. Using R-DeeP, proteins can be classified as RNA-dependent if their interactome is dependent on RNA. R-DeeP thus offers an independent method that avoids potential biases from pulldown or separation based on physicochemical properties while offering at the same time quantitative information on the RNA-dependent fraction of the protein. Additionally, R-DeeP allows reconstructing complexes from proteins shifting out of the same control fraction [30]. While R-DeeP allows determining the RNA-dependence of proteins, it does not provide any information about the RNA binding sites. 

The R-DeeP screen in A549 cells found 1189 RNA-dependent proteins, while 1894 proteins previously linked to RNA in at least one out of 43 human proteome-wide studies did not show significant RNA dependence. One reason for this could be the loss of weak interactions during long centrifugation times or during cellular lysate preparation using detergents [49], while other RBP candidates could be false-positives from previous studies especially if they were found only in very few studies and had a low RBP2GO score [29]. Notably, the non-confirmed RBP candidates from previous studies had a significantly lower RBP2GO score and RBD content than the shifting RNA-binding proteins. In addition, the definition of shifting required a minimum shifting distance of at least one fraction, so that proteins with very small size differences in the presence or absence of RNA could be missed [30].

Interestingly, we noticed that DOCK5 shifted to its monomeric size after RNase treatment, while ELMO2 and ABRAXAS1 remained in a complex larger than their respective monomeric sizes. This illustrates that proteins could be completely released from an RNA-dependent complex (DOCK5) or that they could remain in a complex via protein–protein interactions such as oligomerization of the protein.

Out of the 3743 proteins detected, we quantified 1525 left shifts and 241 right shifts. Left shifts toward lower molecular weights indicate the loss of an interaction partner in the absence of RNA. Given the established functions of RNAs as protein interactors, scaffolds or docking platforms for proteins [31,61] as well as the fact that RNase treatment leads to a loss of the RNA from the complex also decreasing its apparent molecular weight, we expected to see this majority of left shifts. In contrast, right shifts indicate the less frequent gain of new protein interactors upon RNase treatment, e.g., hypothetically by an increased accessibility of interaction regions otherwise occupied by RNA [62]. 

As any method using cellular extracts, artificial interactions could be possible between proteins that do not necessarily take place in a cell [30]. However, the analysis of the RBP2GO score, enrichment of RBDs and IDRs between shifting vs. non-shifting proteins confirms a strong and specific enrichment of RNA-binding proteins in the shifting proteins [29,48,51,52,63]. In turn, the loss of weak or transient interactions may be possible during cell lysis which would lead to false-negative results since proteins would already dissociate in the control conditions.

From the group of newly identified RNA-dependent proteins, we have exemplarily validated the RNA dependence of three proteins. Using the iCLIP2 technique, we further showed the direct RNA binding of DOCK5 and ABRAXAS1, while no suitable antibody for immunoprecipitation was found for the third protein ELMO2. Notably, these proteins are all involved in cancer progression [54,57,59]. Moreover, the 170 new RBP candidates shifting in A549 cells open up opportunities for future research to understand and discover new RNP complexes and their functions in lung cancer.

Furthermore, the R-DeeP 2.0 database contains the complete datasets of RNA-dependent proteins from both HeLa S3 and A549 cells (Figure 7). It allows searches for individual proteins or batch searches for protein lists and displays information about shifts and peaks as well as provides graphical representations of protein profiles and offers download options for data and figures. It also contains orthogonal information on each protein. The database is directly linked to multiple resources that provide useful information about the protein such as UniProt, CORUM, STRING and RBP2GO [29,64,65,66]. We anticipate that the facilitated access to RBP knowledge will support the development of the field of RNA and RBP biology in lung cancer.

## 5. Conclusions

In summary, this R-DeeP screen provides a comprehensive landscape of RNA-dependent proteins in lung cancer cells and identifies novel potential RBP candidates as well as offers an advanced database for RNA-dependent proteins combined with orthogonal data on the proteins and protein complexes. It will stimulate the RNA and RBP research fields with the potential to impact lung cancer research.

## Data Availability

The data presented in this study are publicly available in the R-DeeP 2.0 database at https://R-DeeP2.dkfz.de/. All mass spectrometry proteomics datasets of this study are available online at the MassIVE repository http://massive.ucsd.edu/; dataset identifier: MSV000089645.

## Supplementary Materials

The following supporting information can be downloaded at: https://www.mdpi.com/article/10.3390/cancers14246109/s1, File S1: Original blots of Figures S1B,C, S4C–E, S5B, S6C–E.
