# Supplementary material for: Proteome-Wide Identification of RNA-Dependent Proteins in Lung Cancer Cells
Source: Cancers (Basel). 2022 Dec 12;14(24):6109. doi: 10.3390/cancers14246109 (PMC9776756; doi:10.3390/cancers14246109)

Section 3.1: ***Proteome-wide identification of RNA-dependent proteins in lung cancer cells using the R-DeeP approach***

Figure 1B: hnRNPU 120 kDa

Molecular weight marker: — 130 kDa — 100 kDa

Replicate 1: Top blot: RNase treated gradient; Bottom blot: Control gradient

Gradient samples

Loading order: protein ladder, Fraction 1,2,3,4………………………………………..………25

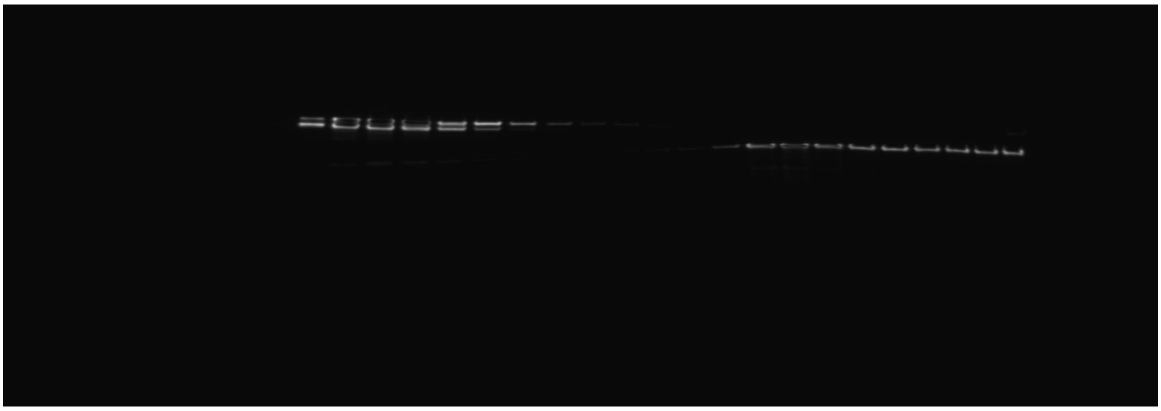

Membrane scan to detect protein ladder

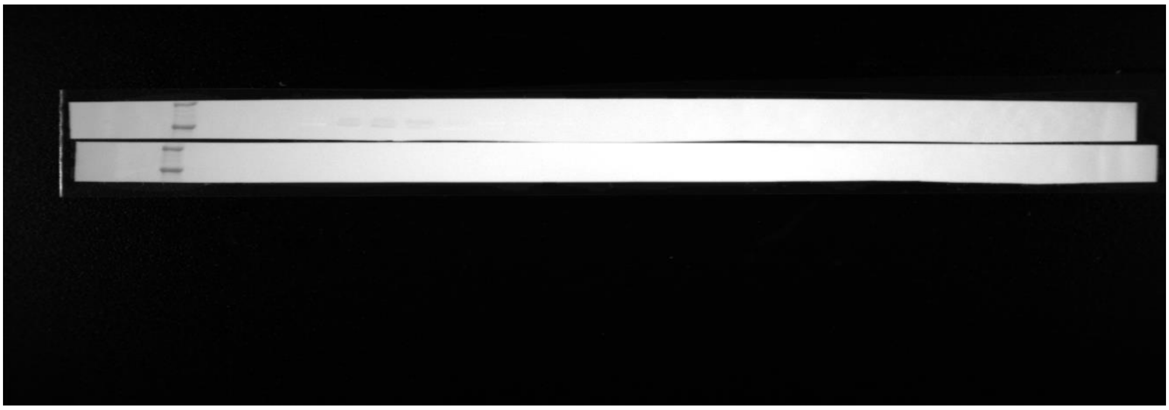

Marker overlay:

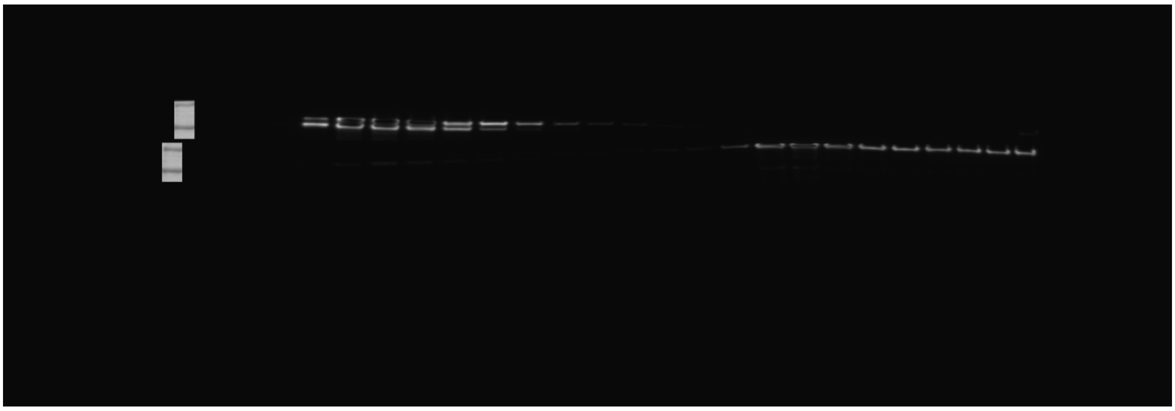

Replicate 2: Top blot: Control gradient ; Bottom blot: RNase treated gradient

Molecular weight marker: 130 kDa
100 kDa

Gradient samples

Loading order: Marker, Fraction 1,2,3,4………………………………………………………………25

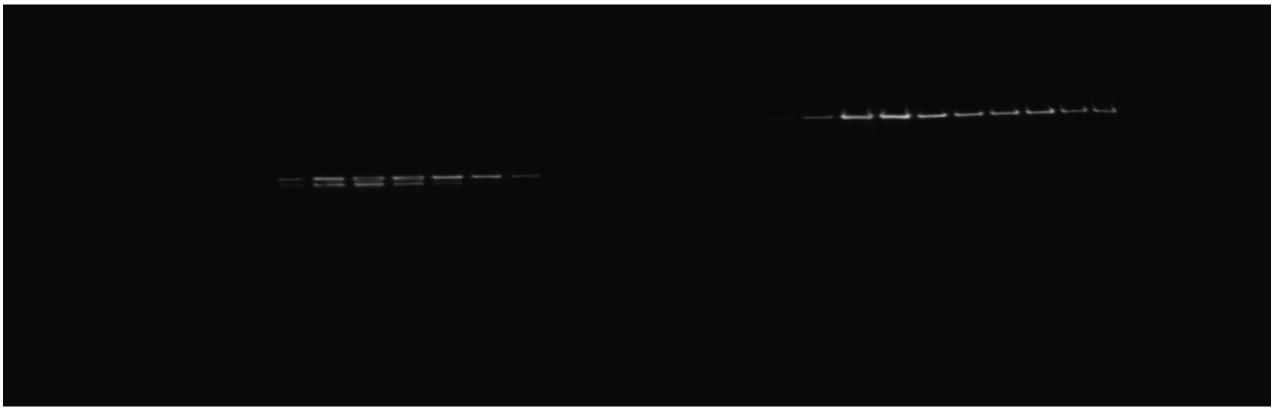

Membrane scan to detect protein ladder

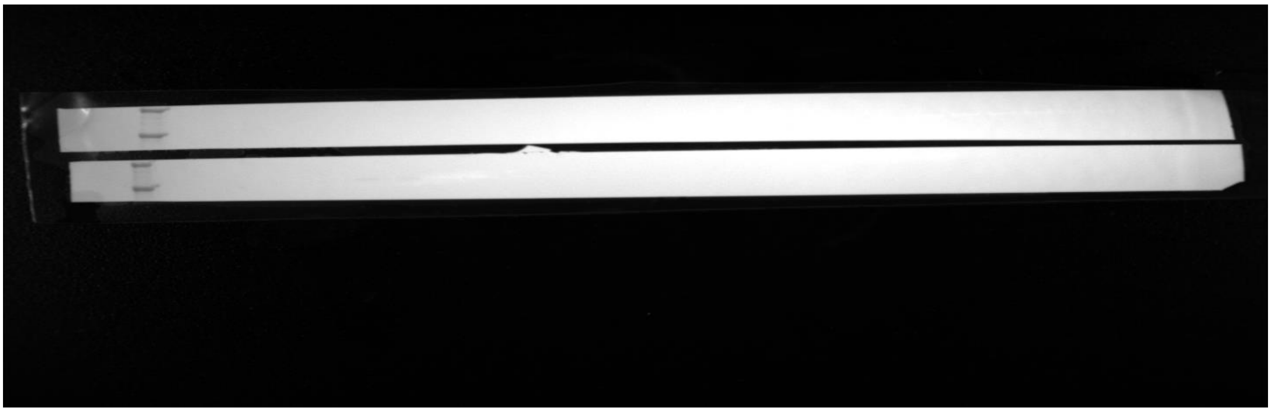

Marker overlay

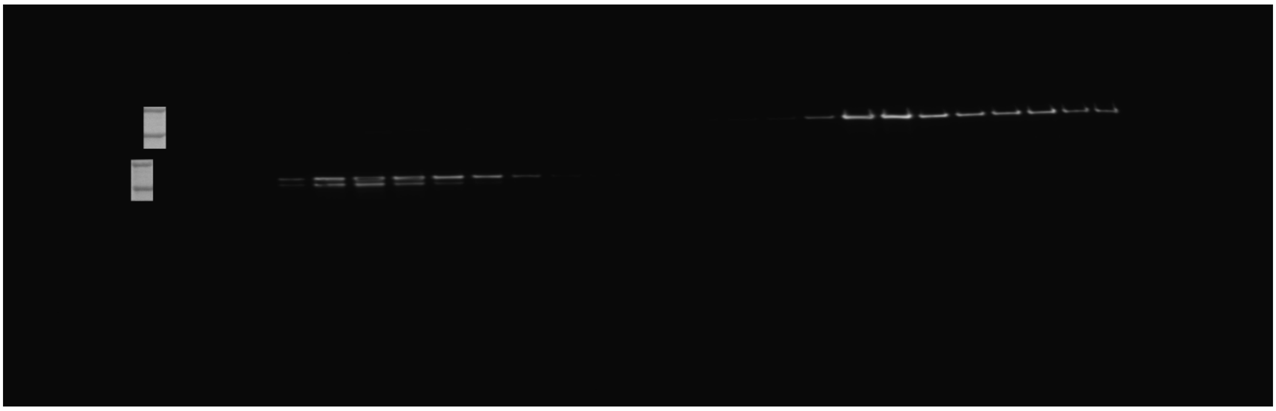

Replicate 3: Top blot: Control gradient; Bottom blot: RNase treated gradient

Molecular weight marker: —— 130 kDa —— 100 kDa

Gradient samples:

Loading order: Marker, Fraction 1,2,3,4……………………………………………………………25

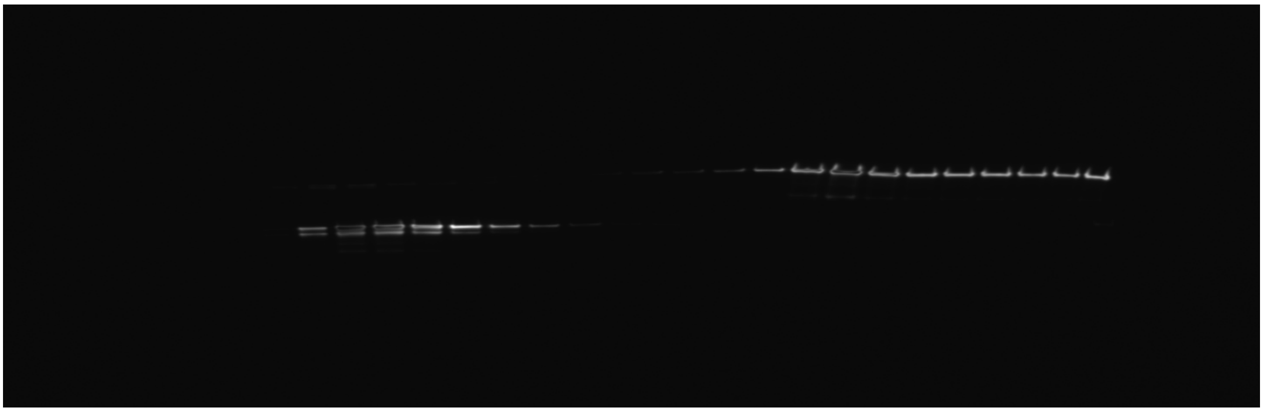

Membrane scan to detect protein ladder

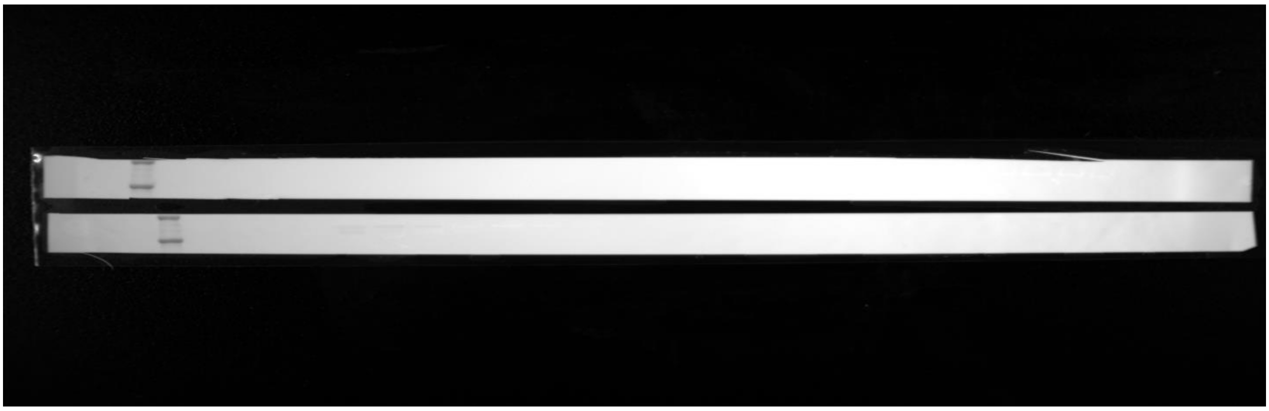

Marker overlay

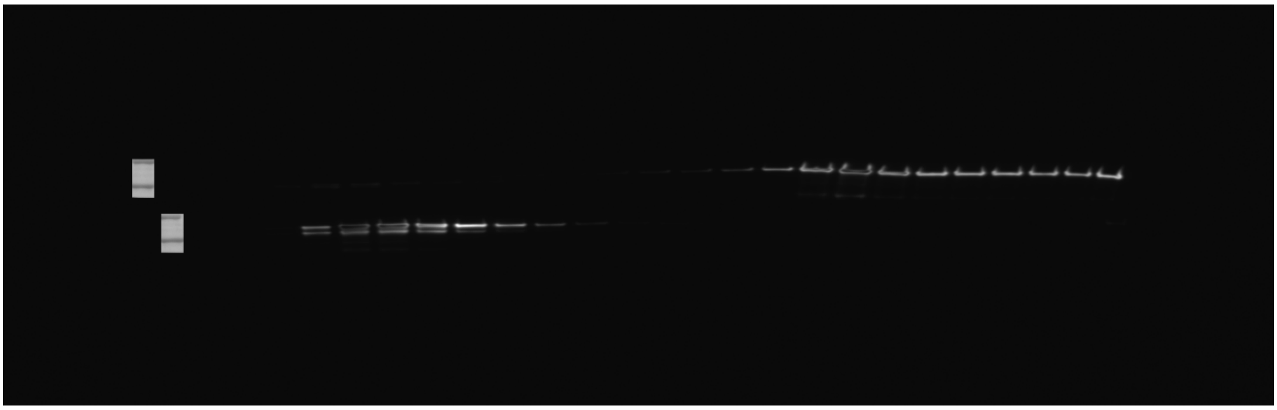

Figure 1C: ASNS 64 kDa

Replicate 1: Top blot left: Control gradient fraction 1-13 , Top blot right: Control gradient 14-25; Bottom blot left: RNase treated gradient 1-13 , Bottom blot right: RNase treated gradient 14-25

The membranes were cut in three portions and stained for different proteins hnRNPU 120 kDa, ASNS 64 kDa and NPM1 37 kDa. The top left, second part of the membrane was flipped upside down during imaging. Hence the ASNS bands are observed above 70 kDa marker in the marker overlay blot.

Molecular weight marker:

— 180 kDa
— 130 kDa
— 100 kDa
— 70 kDa
— 55 kDa
— 40 kDa
— 35 kDa
— 25 kDa

Gradient samples

Loading order: Protein ladder, Fraction 1,2,3,4……13 14,15,16…………………25

ASNS
ASNS

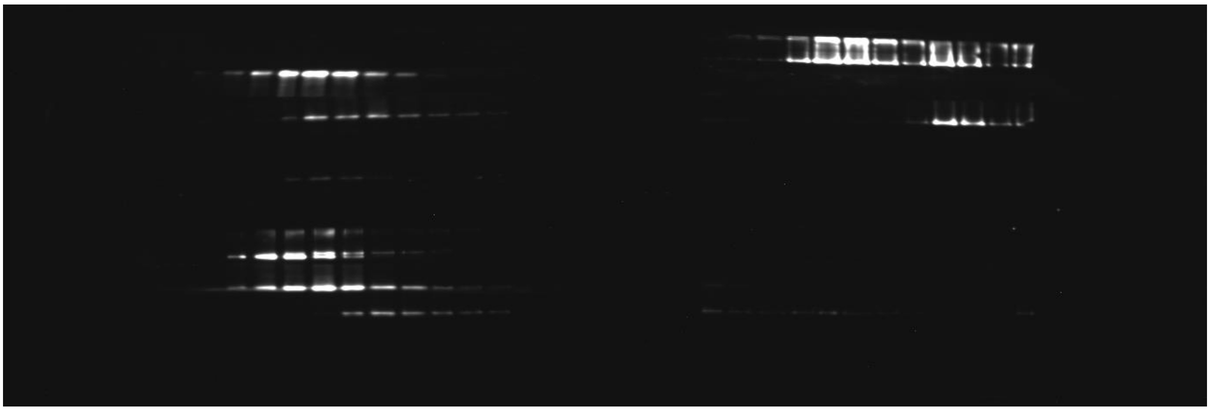

Membrane scan to detect protein ladder

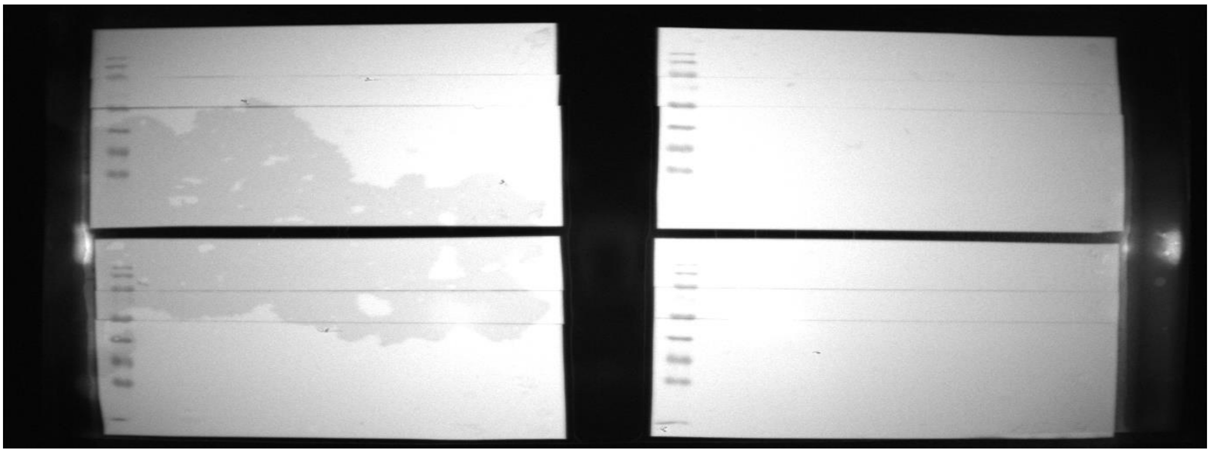

Marker overlay

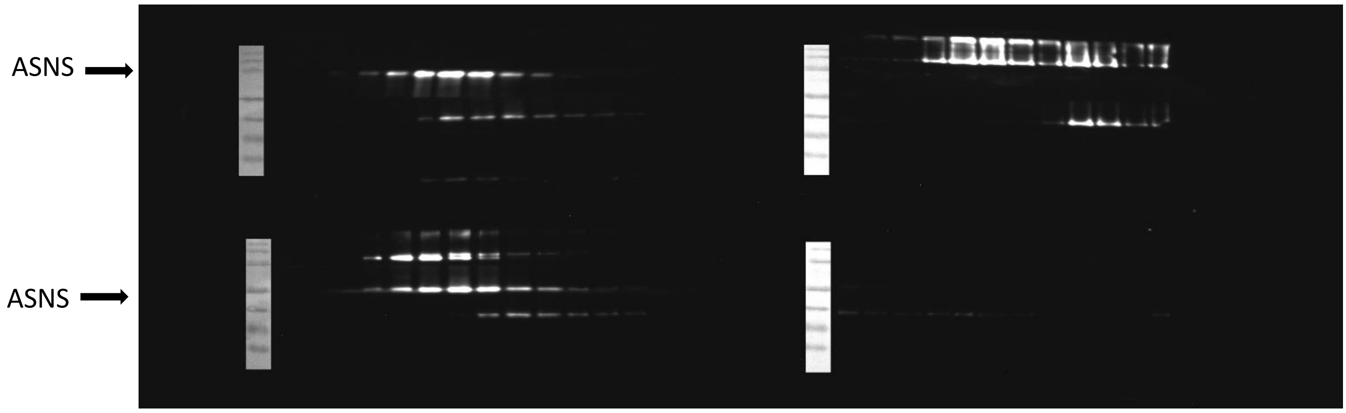

Replicate 2: Top blot left: Control gradient fraction 1-13 , Top blot right: Control gradient 14-25; Bottom blot left: RNase treated gradient 1-13 , Bottom blot right: RNase treated gradient 14-25

Molecular weight marker:
- 180 kDa
- 130 kDa
- 100 kDa
- 70 kDa
- 55 kDa
- 40 kDa
- 35 kDa
- 25 kDa

Gradient samples

Loading order: Protein ladder, Fraction 1,2,3,4……13 14,15,16………….………25

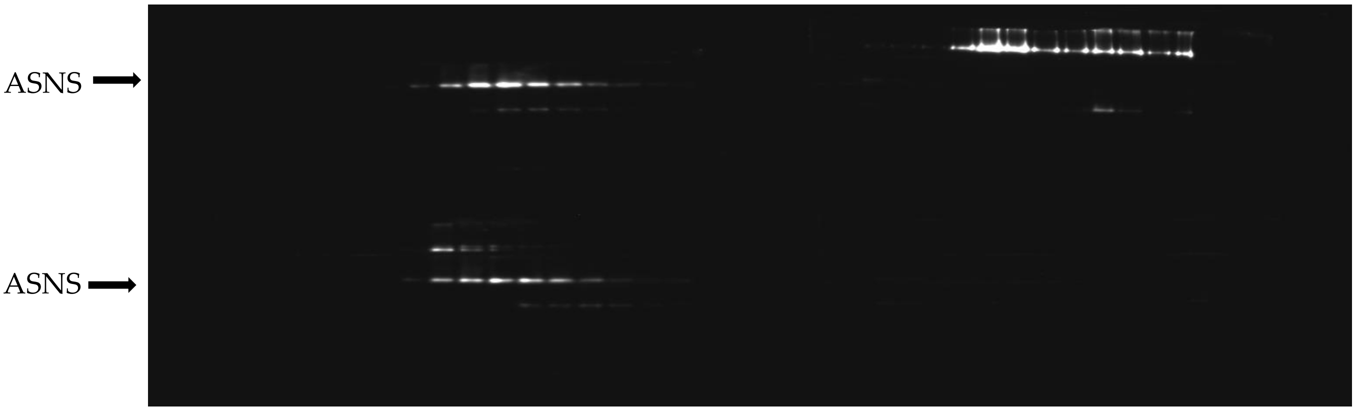

Membrane scan to detect protein ladder

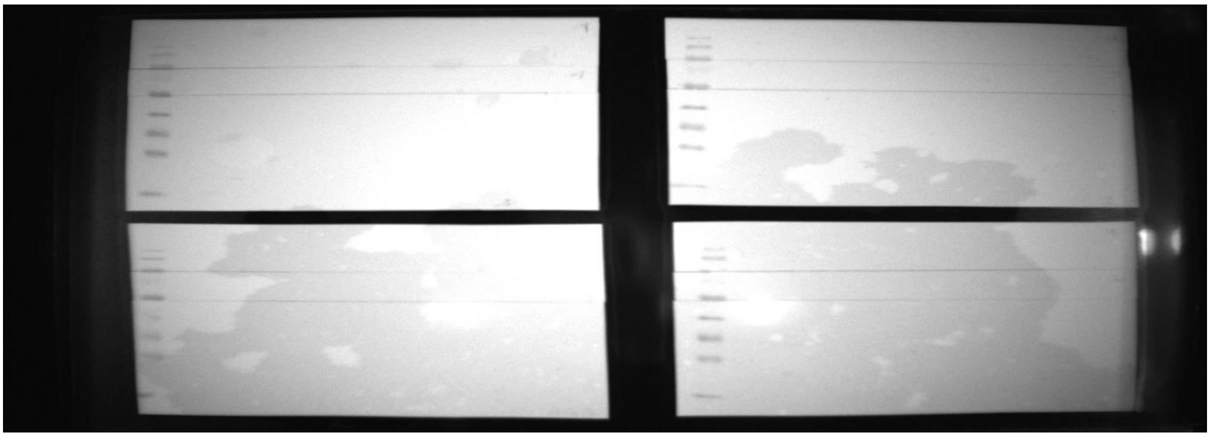

Marker overlay

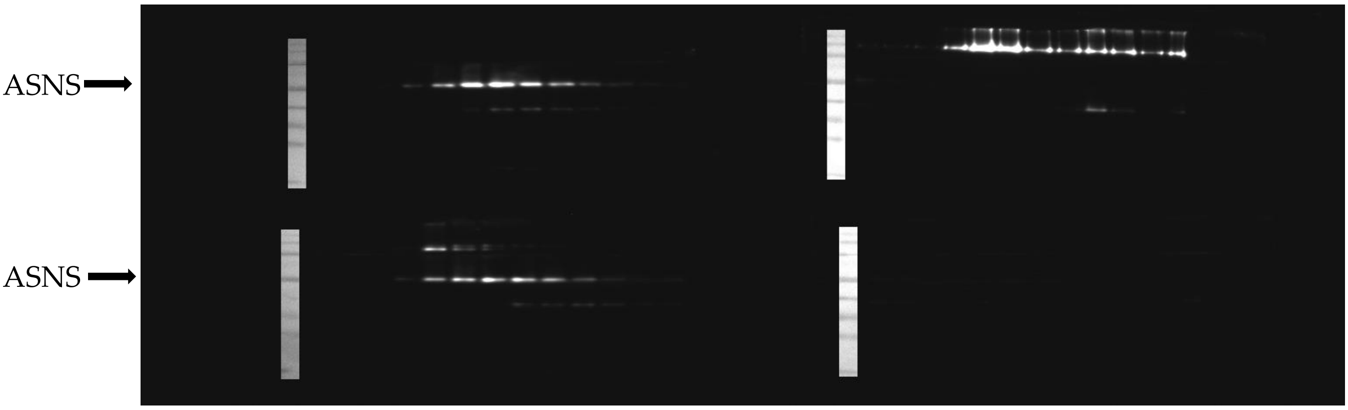

Replicate 3: Top blot left: Control gradient fraction 1-13 , Top blot right: Control gradient 14-25; Bottom blot left: RNase treated gradient 1-13 , Bottom blot right: RNase treated gradient 14-25

Molecular weight marker:
- 180 kDa
- 130 kDa
- 100 kDa
- 70 kDa
- 55 kDa
- 40 kDa
- 35 kDa
- 25 kDa

Gradient samples

Loading order: Protein ladder, Fraction 1,2,3,4……13 14,15,16…………..………25

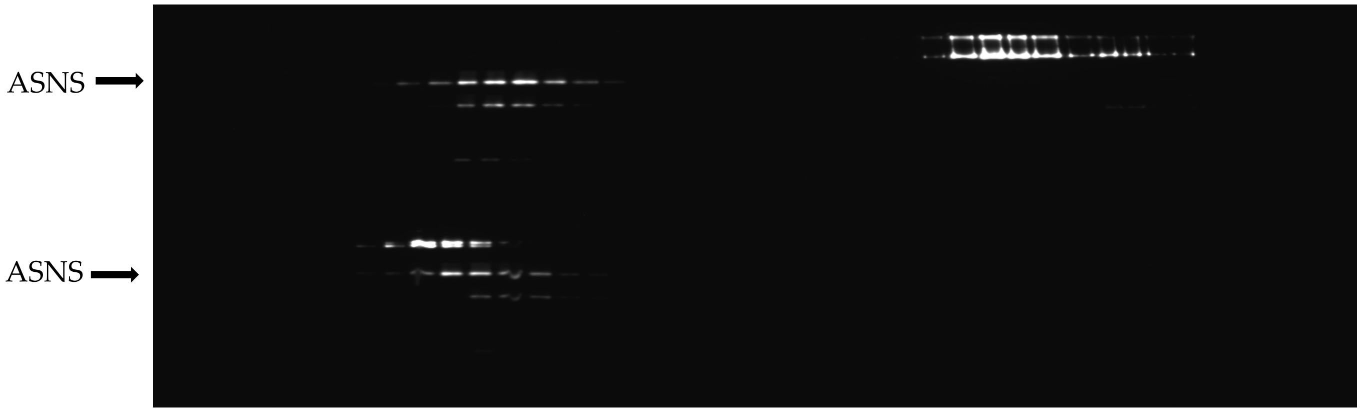

Membrane scan to detect protein ladder

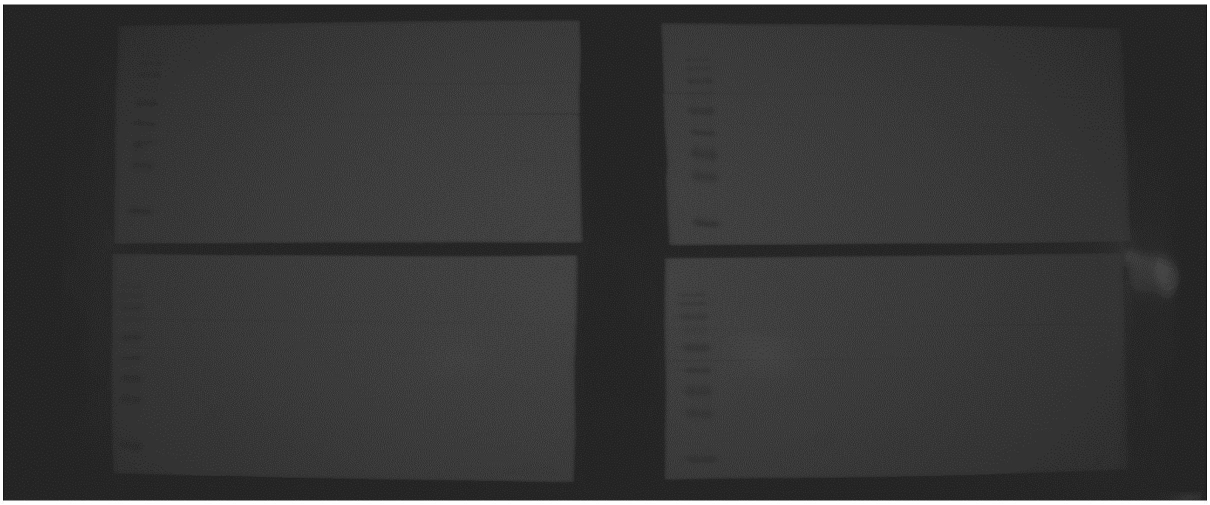

Marker overlay

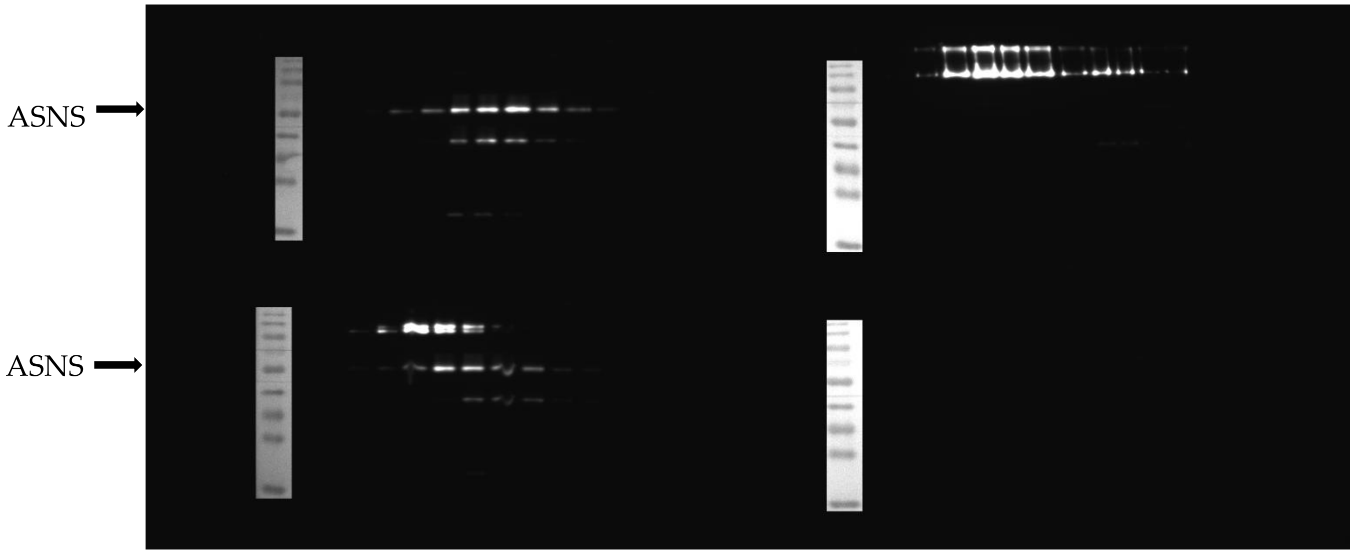

Section 3.4: Validation of DOCK5 as RNA-dependent protein

Figure 4C: DOCK5 215 kDa protein

Replicate 1: Top blot: Control gradient; Bottom blot: RNase treated gradient

Molecular weight marker: —— 250 kDa

Gradient samples

Loading order: protein ladder, Fraction 1,2,3,4……………………………………..………25

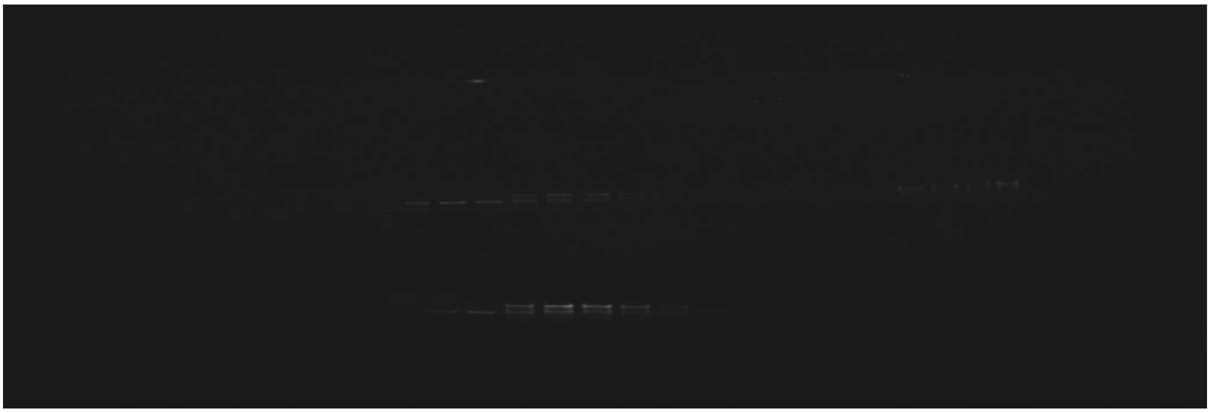

Membrane scan to detect protein ladder

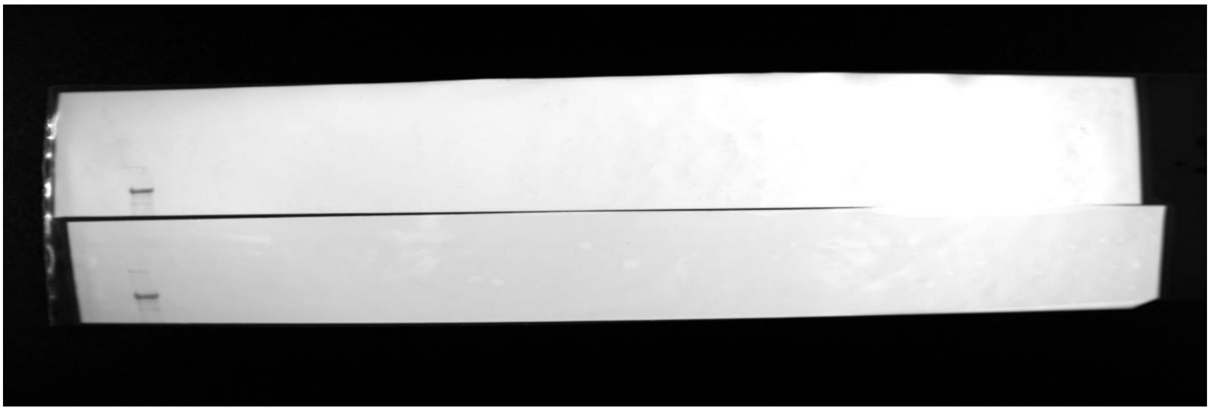

Marker overlay

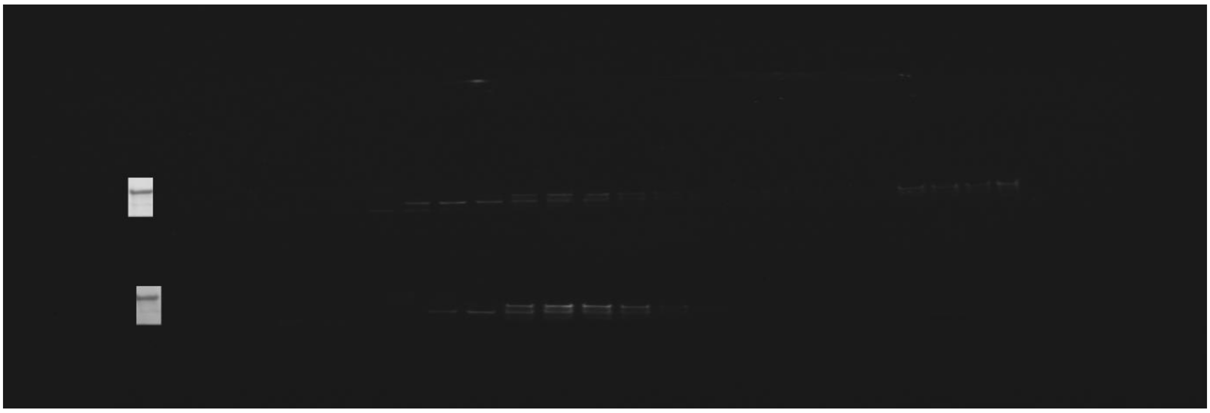

Replicate 2: Top blot: Control gradient; Bottom blot: RNase treated gradient

Molecular weight marker: —— 250 kDa

Gradient samples

Loading order: protein ladder, Fraction 1,2,3,4………………………………….………25

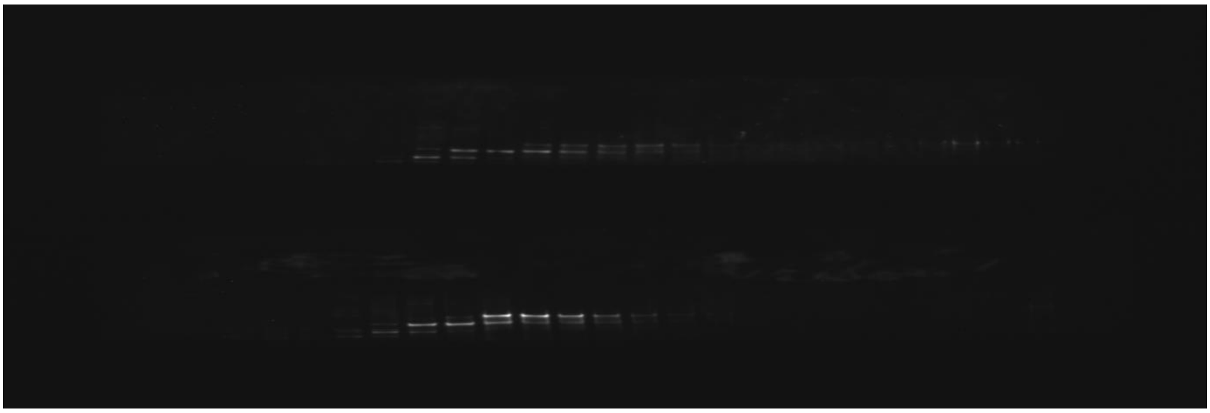

Membrane scan to detect protein ladder

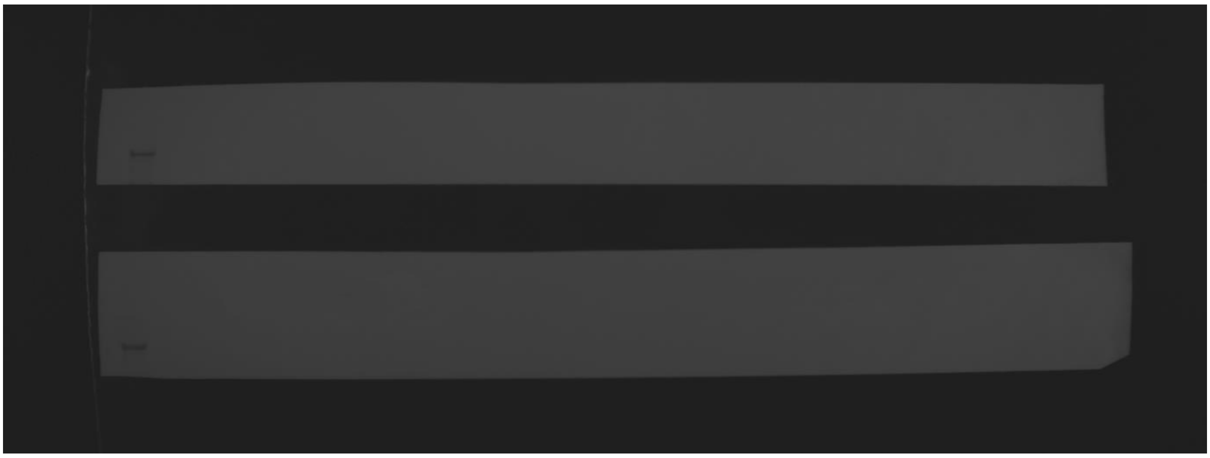

Marker overlay

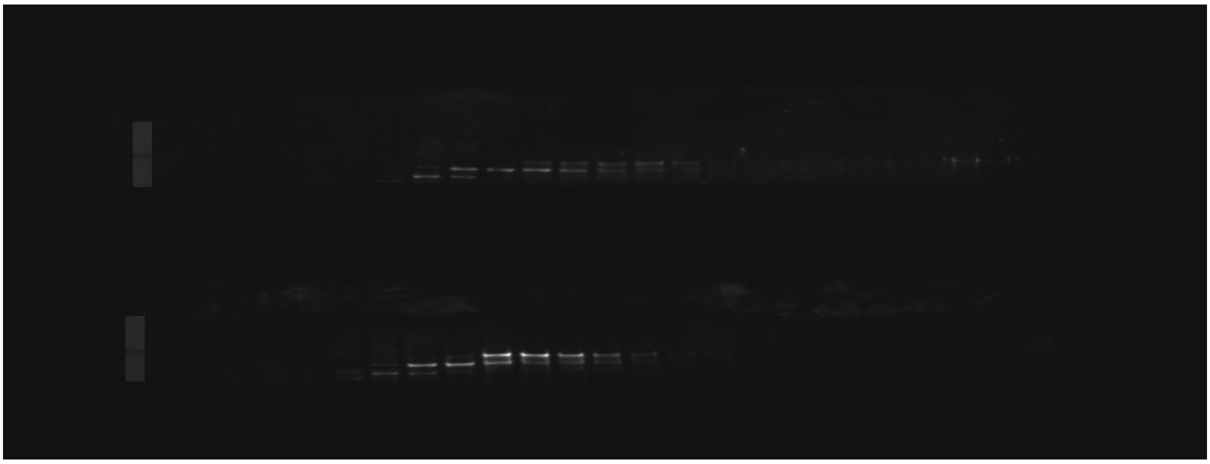

Replicate 3: Top blot: Control gradient; Bottom blot: RNase treated gradient

Molecular weight marker: —— 250 kDa

Gradient samples

Loading order: protein ladder, Fraction 1,2,3,4…………………………………..………25

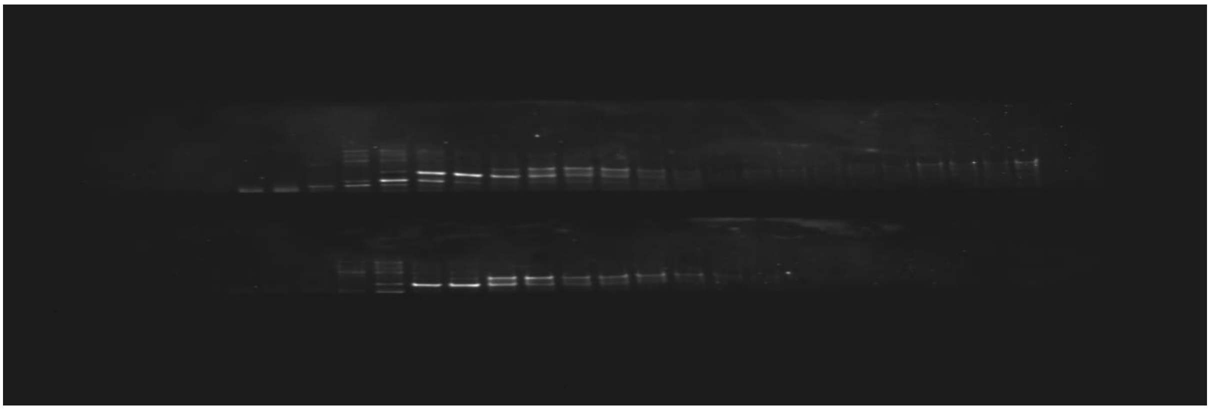

Membrane scan to detect protein ladder

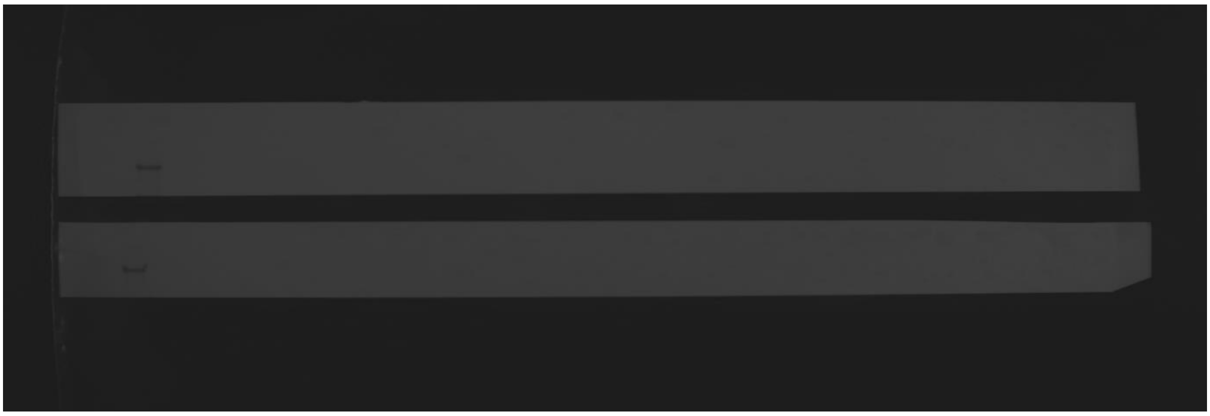

Marker overlay

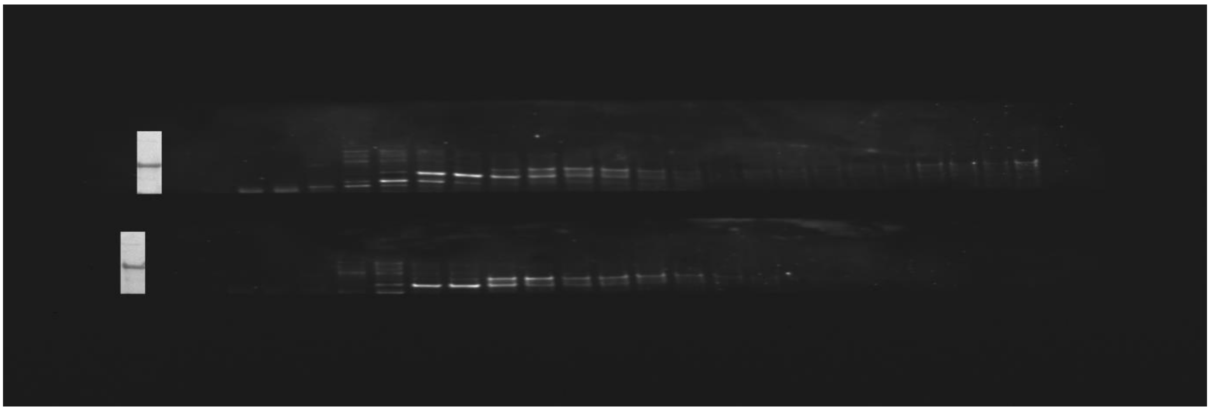

Figure 4D: Direct RNA binding of DOCK5

Autoradiography [32P]

Replicate 1:

UV Crosslinked, 254nm, 200mJ/cm^2 **Non-Crosslinked**

IgG **DOCK5**

RNase I dilution **1:5** **1:5** **1:50** **1:500** **1:5**

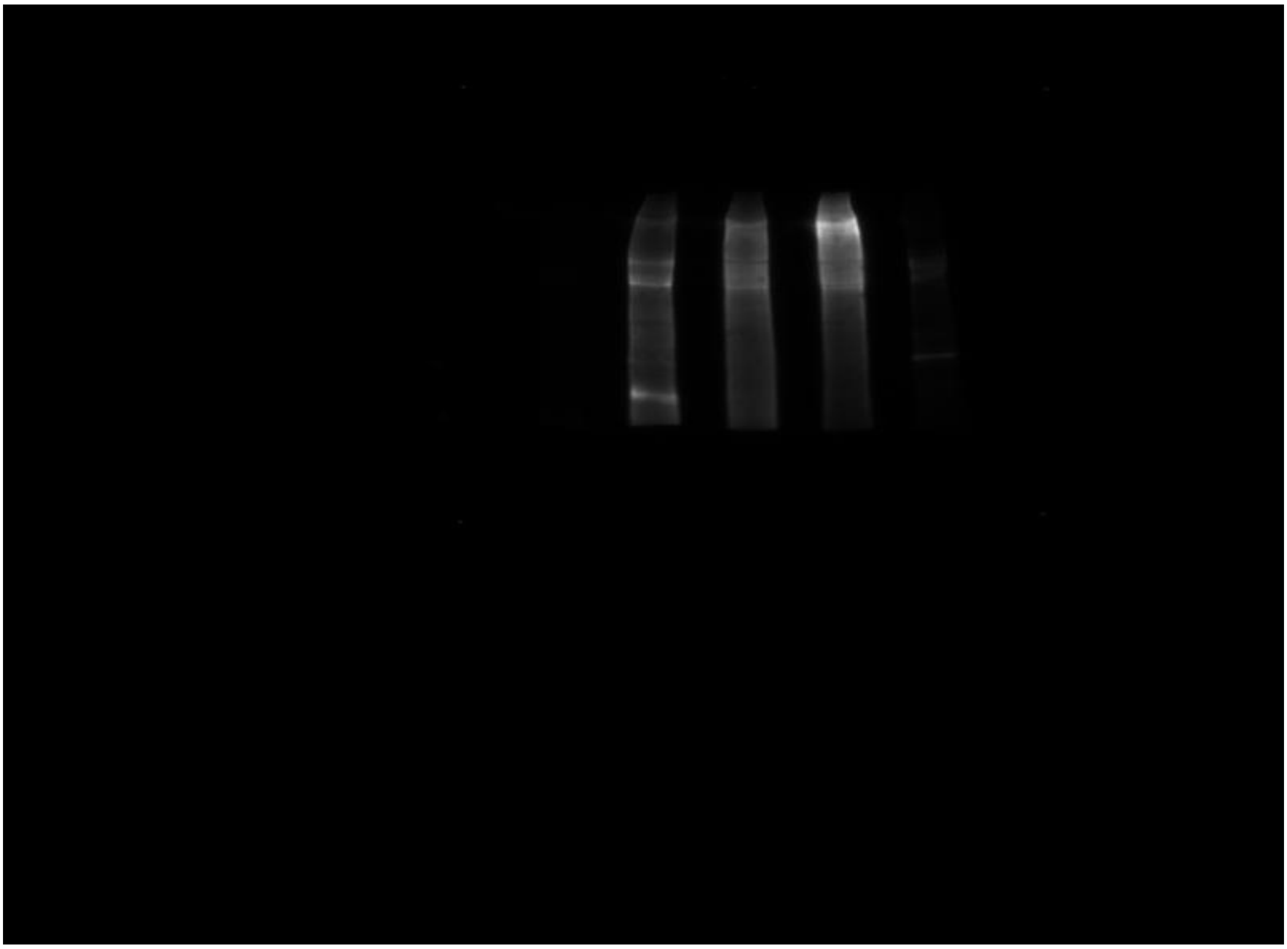

Replicate 2:

UV Crosslinked, 254nm, 200mJ/cm^2 **Non-Crosslinked**

	IgG	DOCK5			
**RNase I dilution**	**1:5**	**1:5**	**1:50**	**1:500**	**1:5**

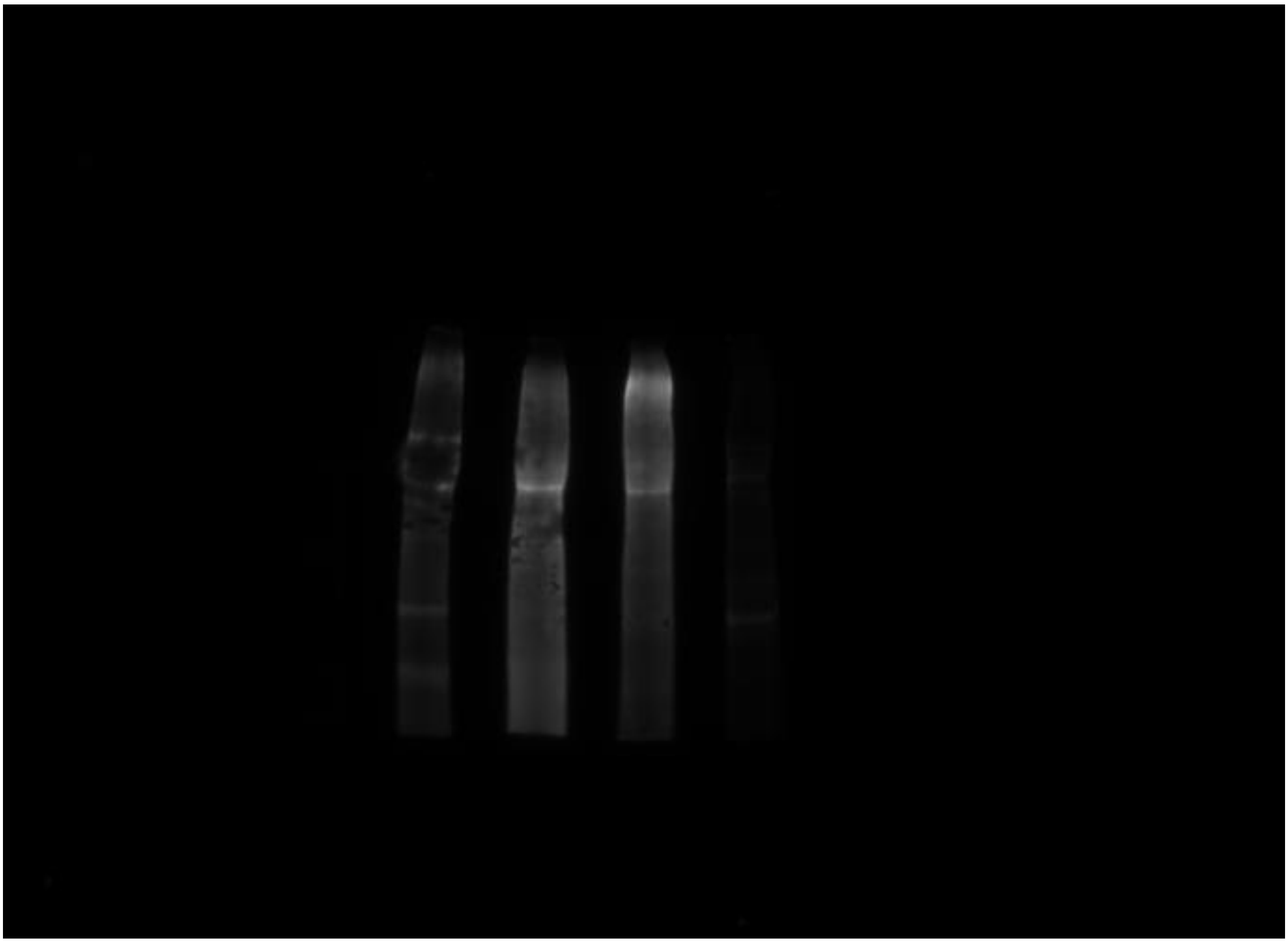

Replicate 3:

UV Crosslinked, 254nm, 200mJ/cm^2 **Non-Crosslinked**

IgG **DOCK5**

**RNase I dilution**	**1:5**	**1:5**	**1:50**	**1:500**	**1:5**

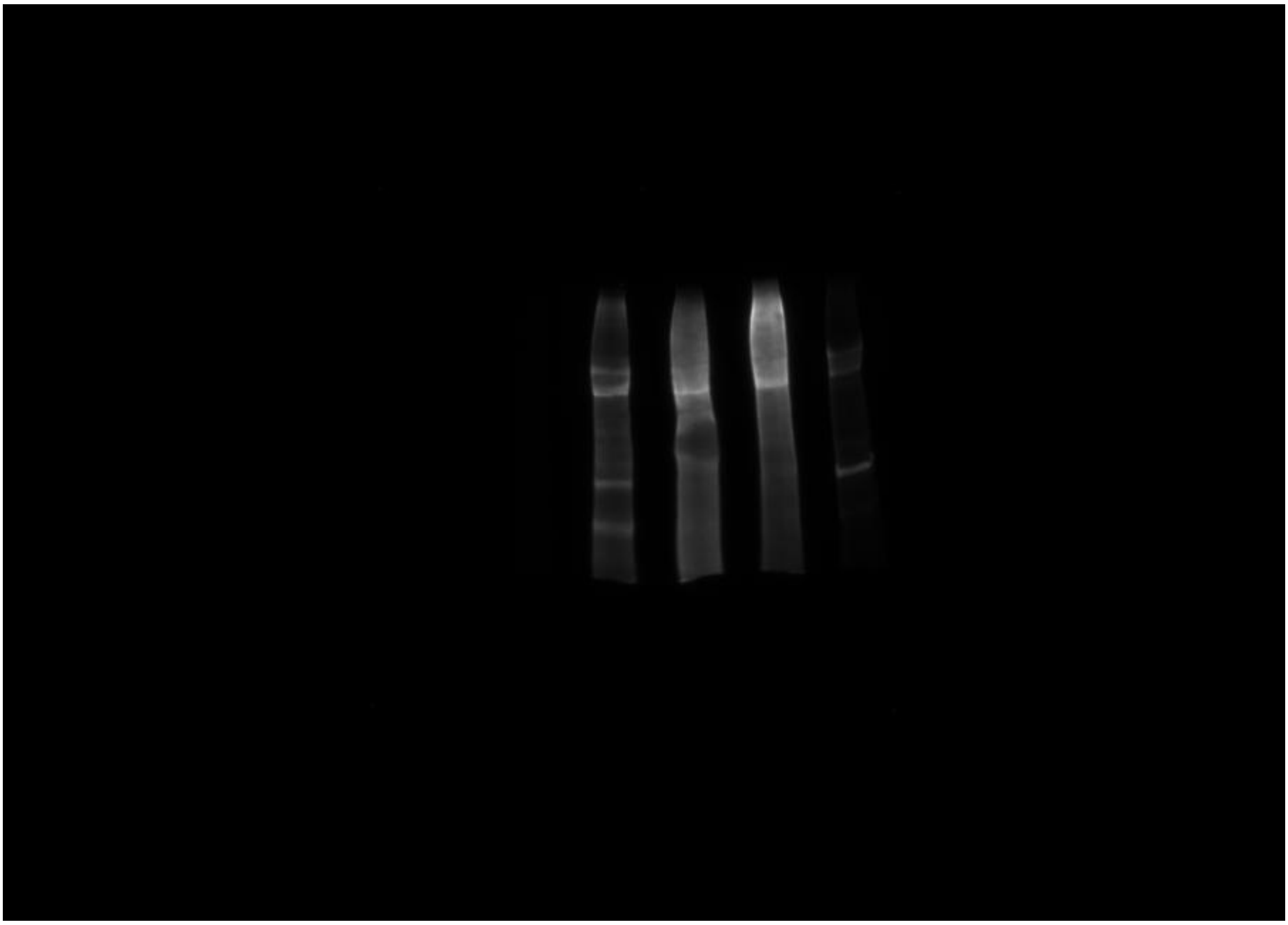

Figure 4E: Immunoprecipitation efficiency of DOCK5 for iCLIP2

Replicate 1:

MW: 215 kDa

Loading order: Marker/Lysate CL, FT IgG 1:5 , IP IgG 1:5, FT DOCK5 1:5, IP DOCK5 1:5, FT DOCK5 1:50, IP DOCK5 1:50, FT DOCK5 1:500, IP DOCK5 1:500, lysate NCL, FT DOCK5 NCL 1:5, IP DOCK5 NCL 1:5 (Lysate/FT:5%) (FT: Flowthrough, CL: Crosslink)

Molecular weight marker:
- 250 kDa
- 130 kDa
- 100 kDa
- 70 kDa
- 55 kDa

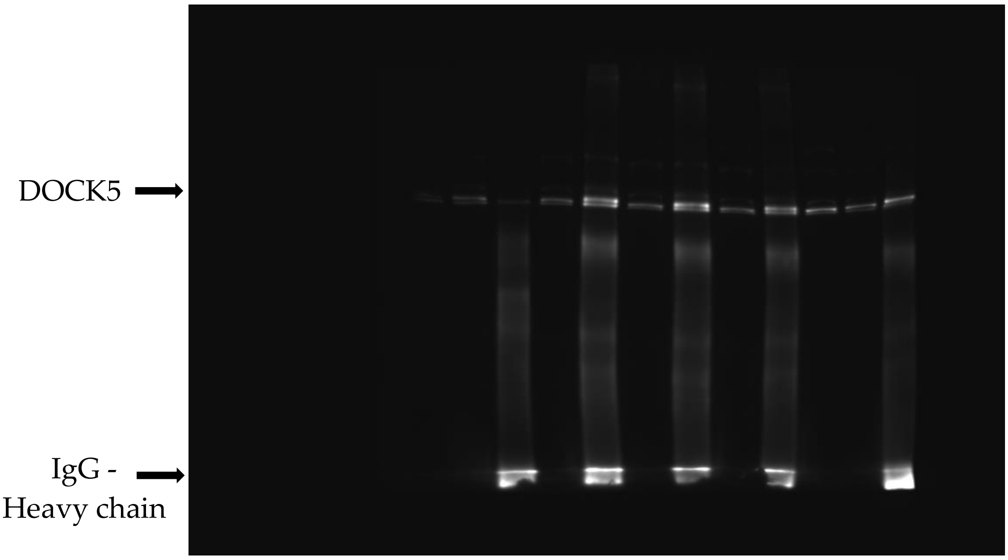

Membrane scan to detect protein ladder

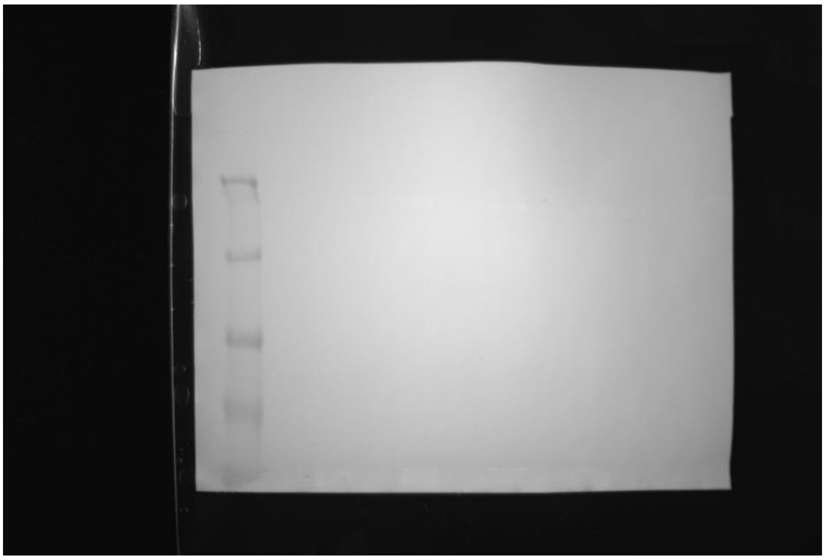

Marker overlay

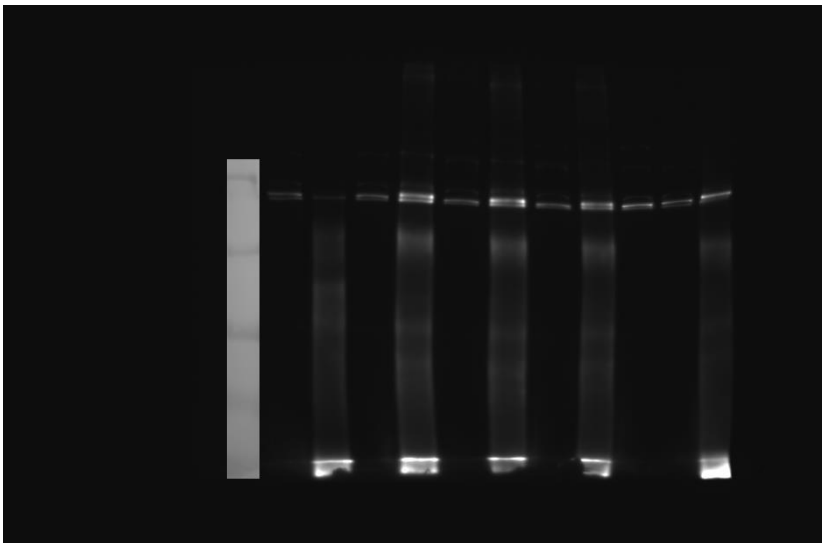

Replicate 2:

MW: 215 kDa

Loading order: Marker/Lysate CL, FT IgG 1:5, IP IgG 1:5, FT DOCK5 1:5, IP DOCK5 1:5, FT DOCK5 1:50, IP DOCK5 1:50, FT DOCK5 1:500, IP DOCK5 1:500, lysate NCL, FT DOCK5 NCL 1:5, IP DOCK5 NCL 1:5 (Lysate/FT:5%)

Molecular weight marker:
- 250 kDa
- 130 kDa
- 100 kDa
- 70 kDa
- 55 kDa

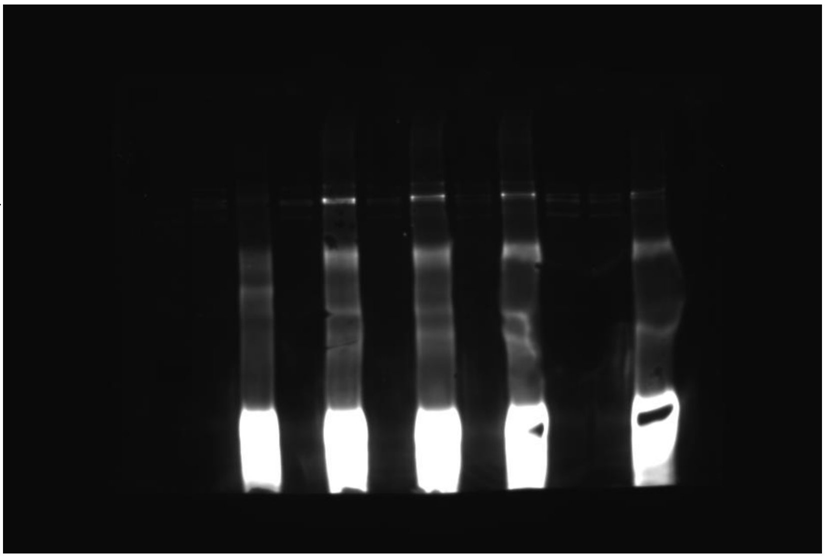

Membrane scan to detect protein ladder:

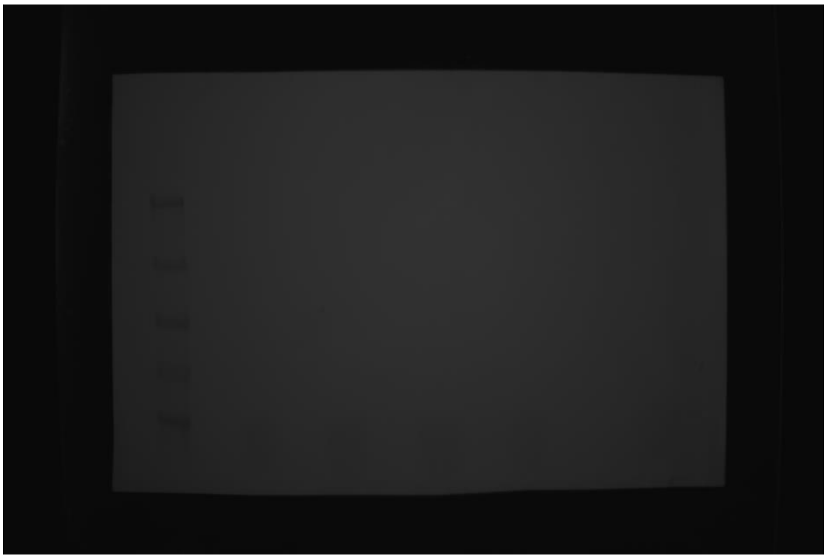

Marker overlay:

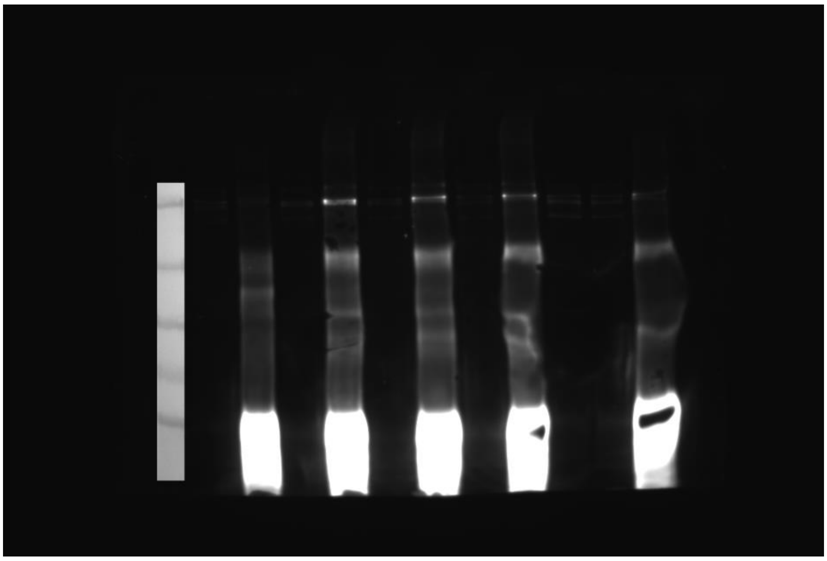

Replicate 3:

MW: 215 kDa

Loading order: Marker/Lysate CL, FT IgG 1:5 , IP IgG 1:5, FT DOCK5 1:5, IP DOCK5 1:5, FT DOCK5 1:50, IP DOCK5 1:50, FT DOCK5 1:500, IP DOCK5 1:500, lysate NCL, FT DOCK5 NCL 1:5, IP DOCK5 NCL 1:5 (Lysate/FT:5%)

Molecular weight marker:

- 250 kDa
- 130 kDa
- 100 kDa
- 70 kDa
- 55 kDa

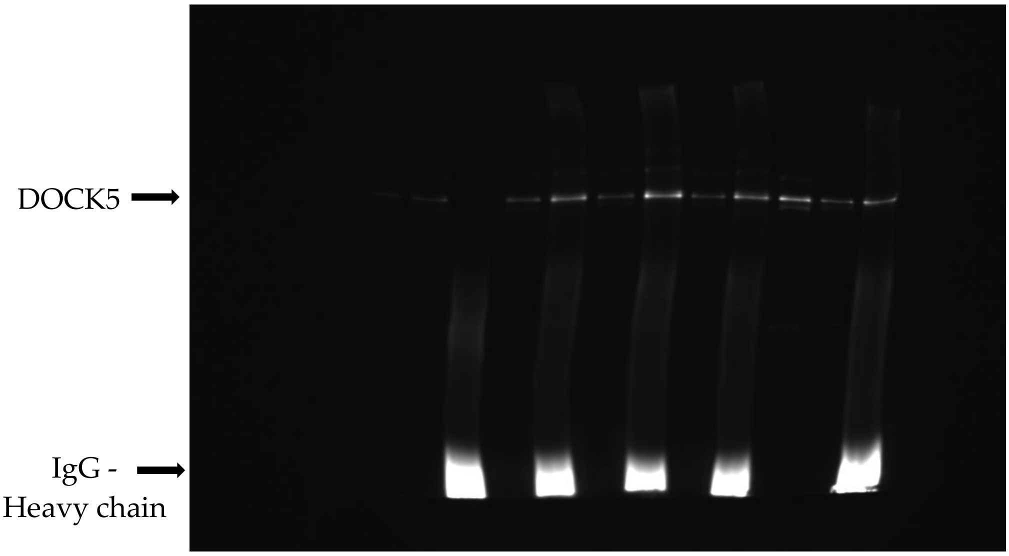

Membrane scan to detect protein ladder

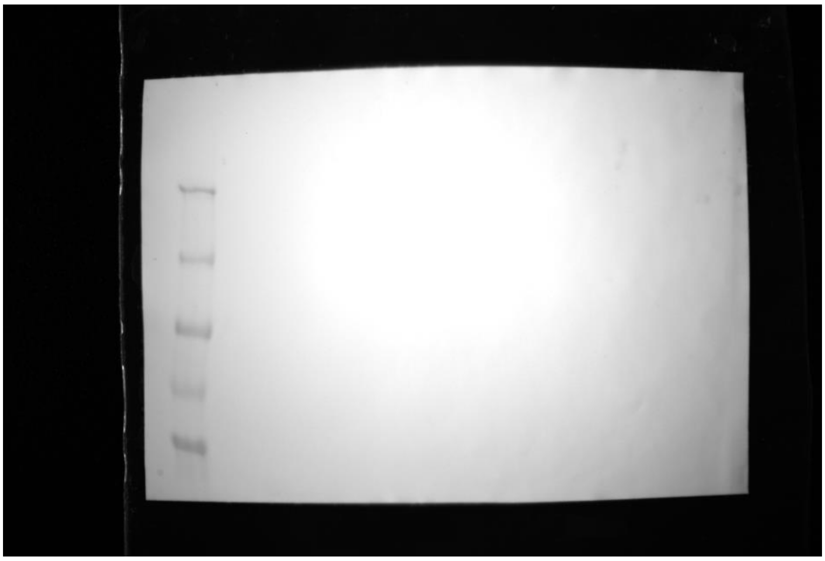

Marker overlay:

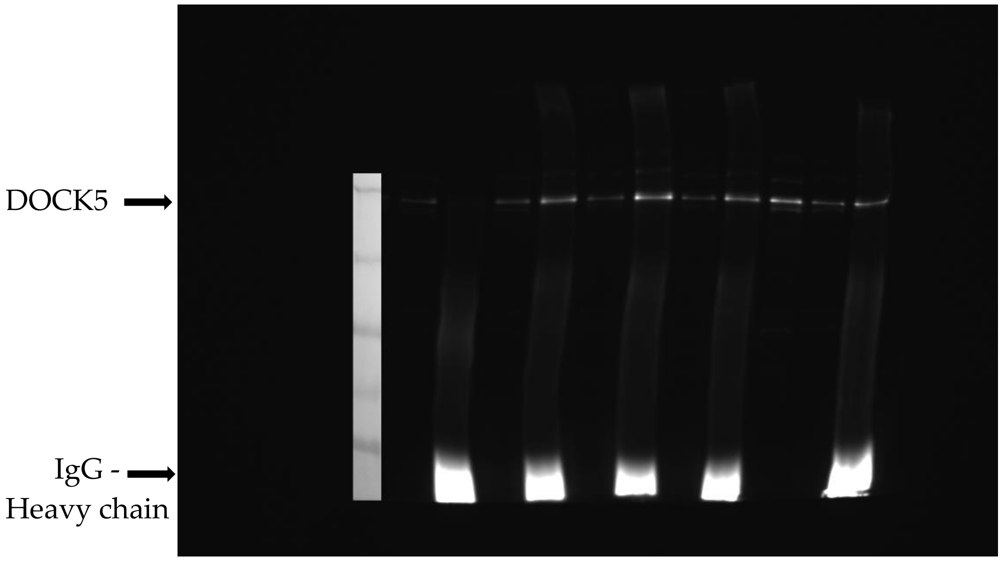

__Section 3.5: Validation of ELMO2 as RNA-dependent protein__

Figure 5B: ELMO2 82 kDa protein

The blot was stained for 2 different proteins ELMO2 84 kDa and FSCN1 55 kDa. The lower band represents FSCN1, and the upper bands represent ELMO2 protein.

Replicate 1: Top blot: Control gradient; Bottom blot: RNase treated gradient

Molecular weight marker: — 100 kDa — 75 kDa — 50 kDa

Gradient samples

Loading order: protein ladder, Fraction 1,2,3,4…………………………………..……….25

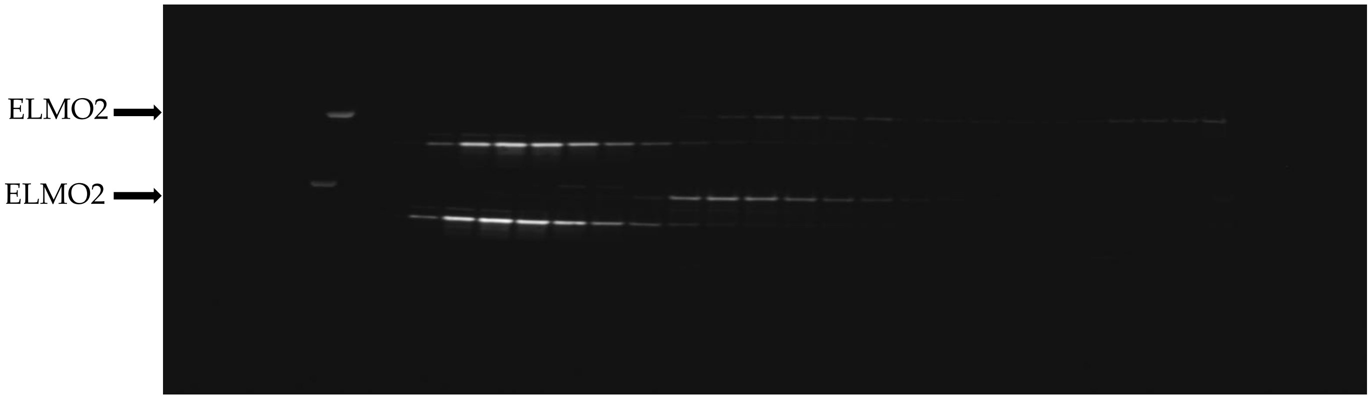

Membrane scan to detect protein ladder

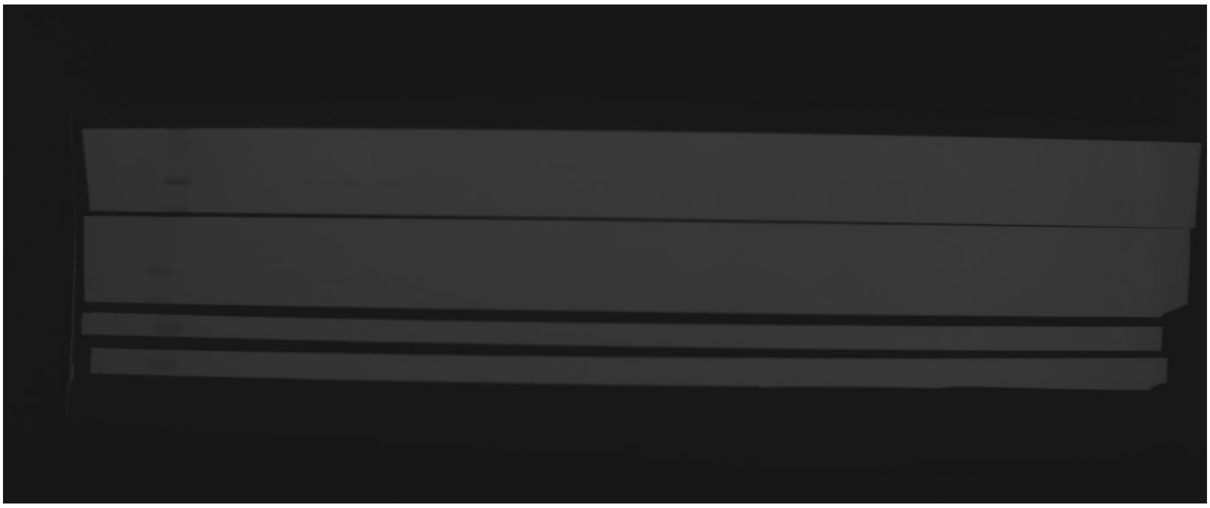

Marker overlay

ELMO2

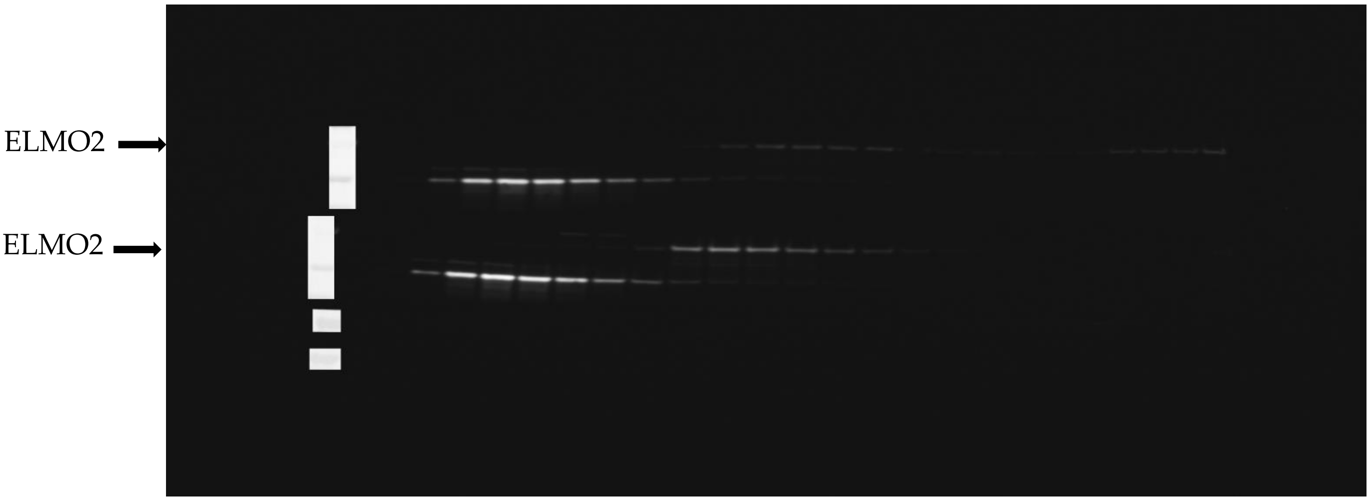

ELMO2

Replicate 2: Top blot: Control gradient; Bottom blot: RNase treated gradient

Molecular weight marker:
130 kDa
100 kDa
75 kDa
50 kDa
37 kDa

Gradient samples

Loading order: protein ladder, Fraction 1,2,3,4………………………………………..………25

ELMO2

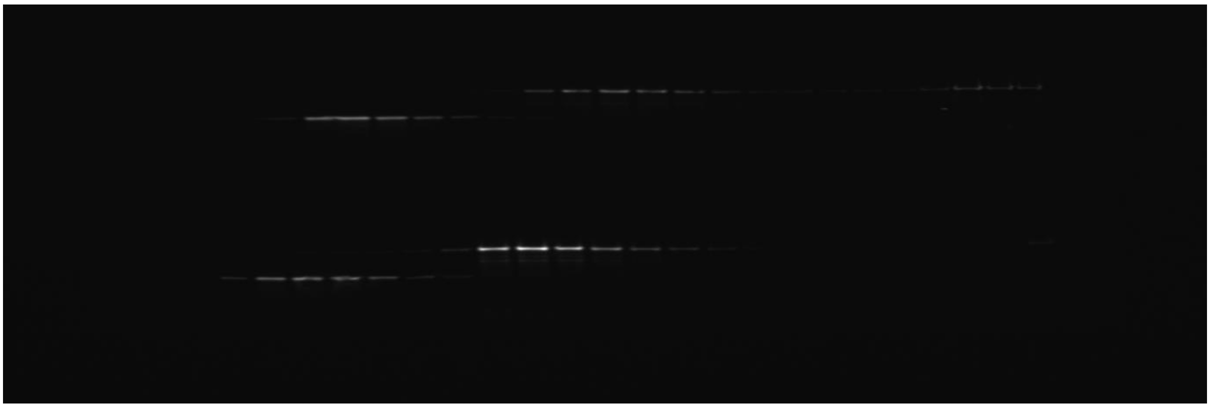

ELMO2

Membrane scan to detect protein ladder

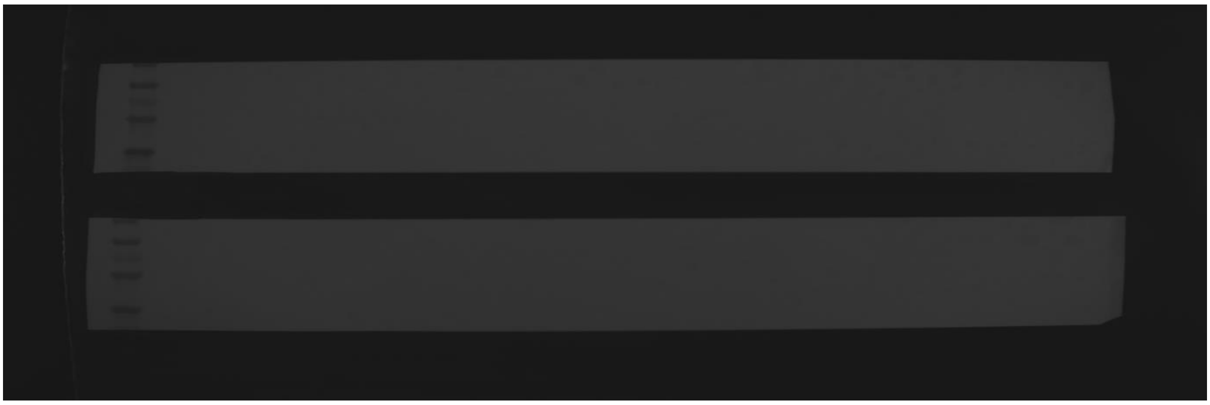

Marker overlay

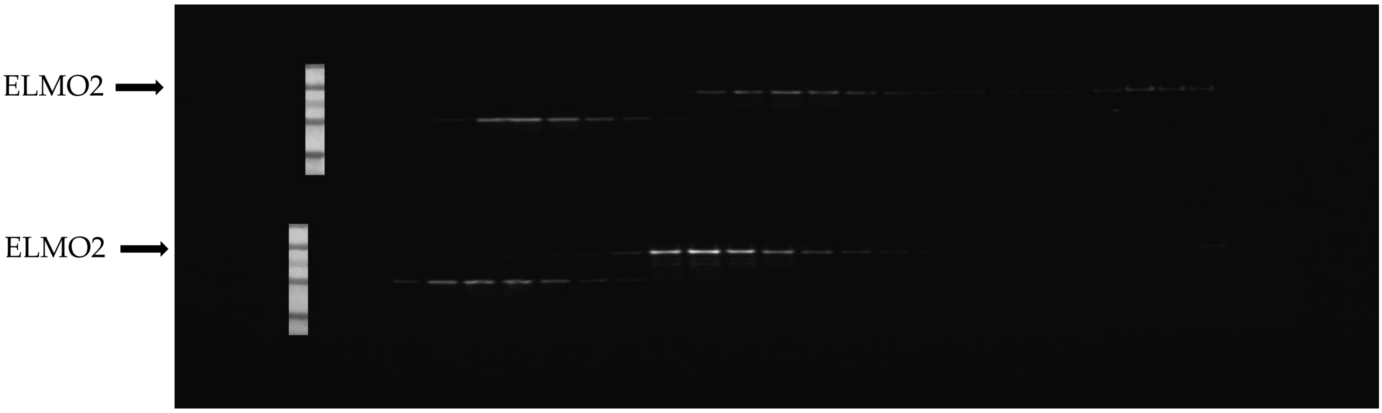

Replicate 3: Top blot: Control gradient; Bottom blot: RNase treated gradient

Molecular weight marker:
- 130 kDa
- 100 kDa
- 75 kDa
- 50 kDa
- 37 kDa

Gradient samples

Loading order: protein ladder, Fraction 1,2,3,4……………………………………..………25

ELMO2

ELMO2

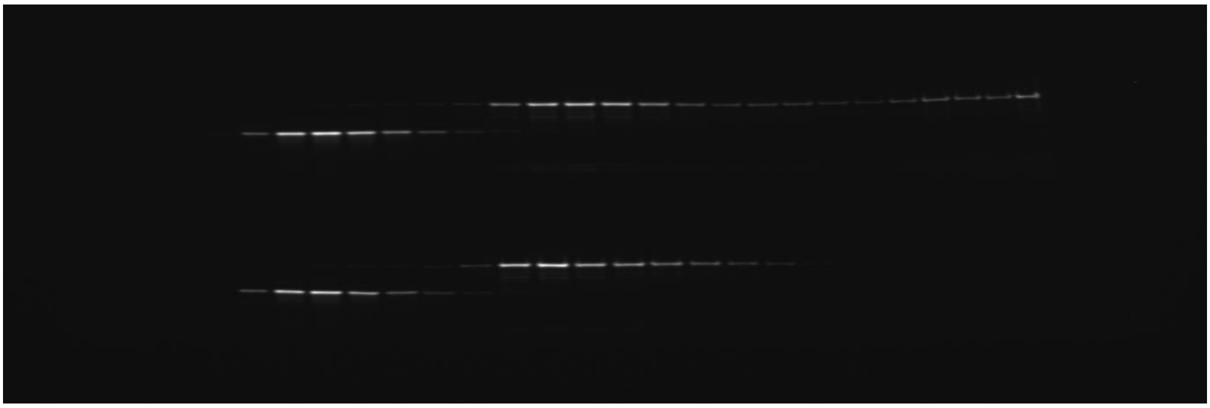

Membrane scan to detect protein ladder

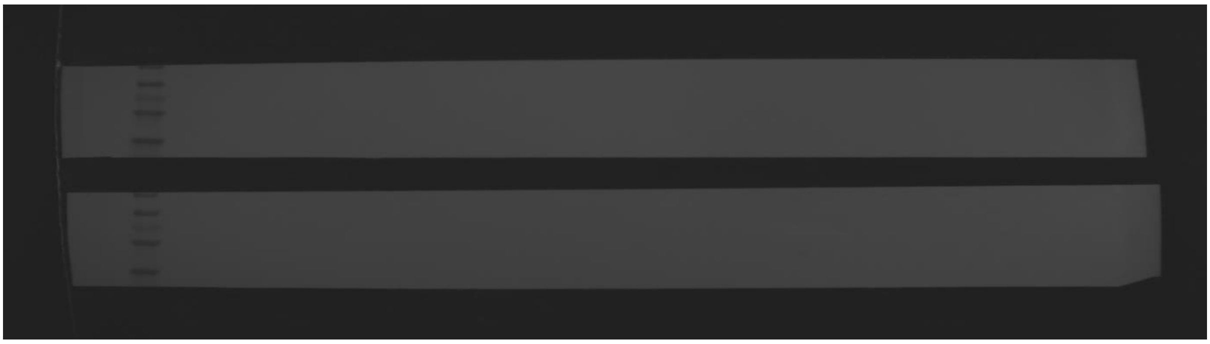

Marker overlay

ELMO2

ELMO2

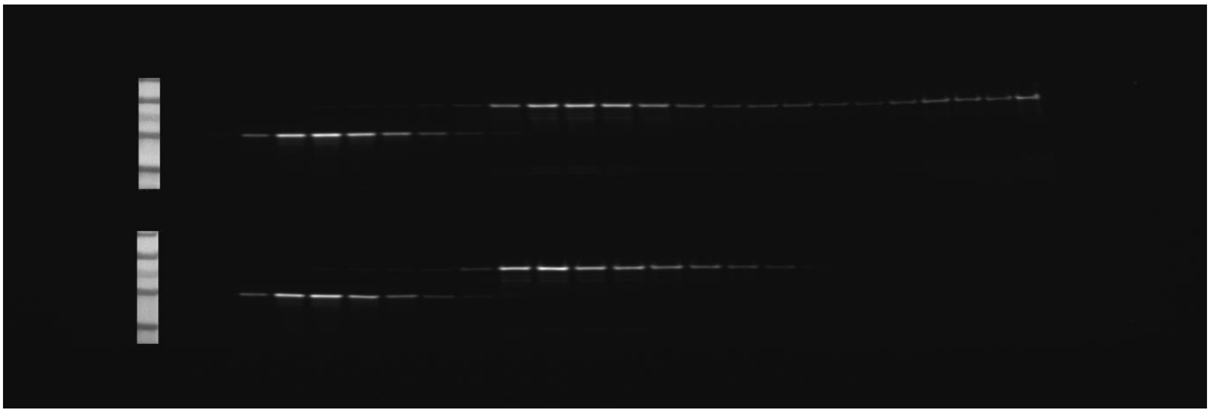

Section 3.6: Validation of ABRAXAS1 as RNA-dependent protein

Figure 6C: ABRAXAS1 47 kDa protein

Replicate 1: Top blot: Control gradient; Bottom blot: RNase treated gradient

Molecular weight marker:
- 50 kDa
- 37 kDa
- 25 kDa
- 20 kDa
- 15 kDa
- 10 kDa

Gradient samples

Loading order: protein ladder, Fraction 1,2,3,4…………………………………...………25

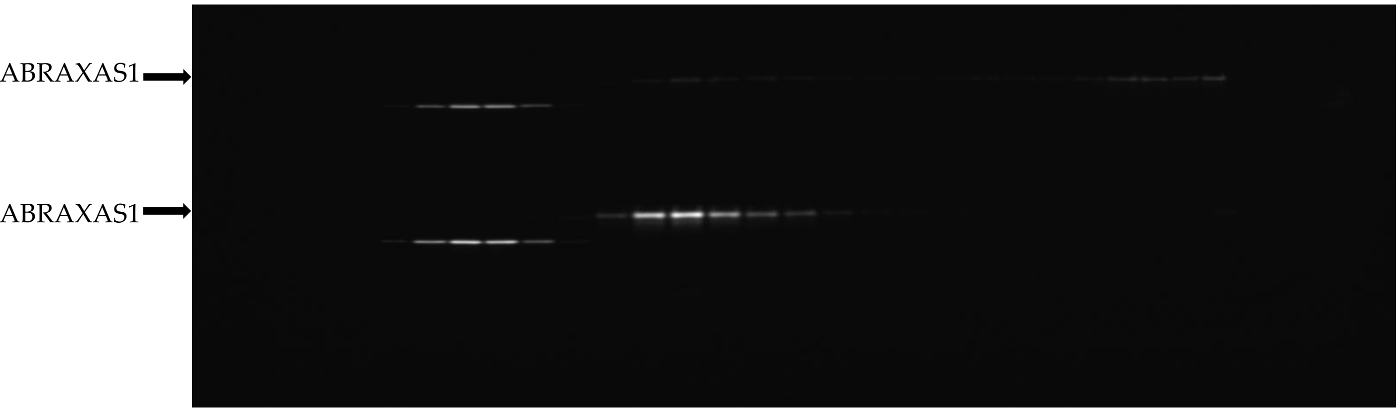

Membrane scan to detect protein ladder

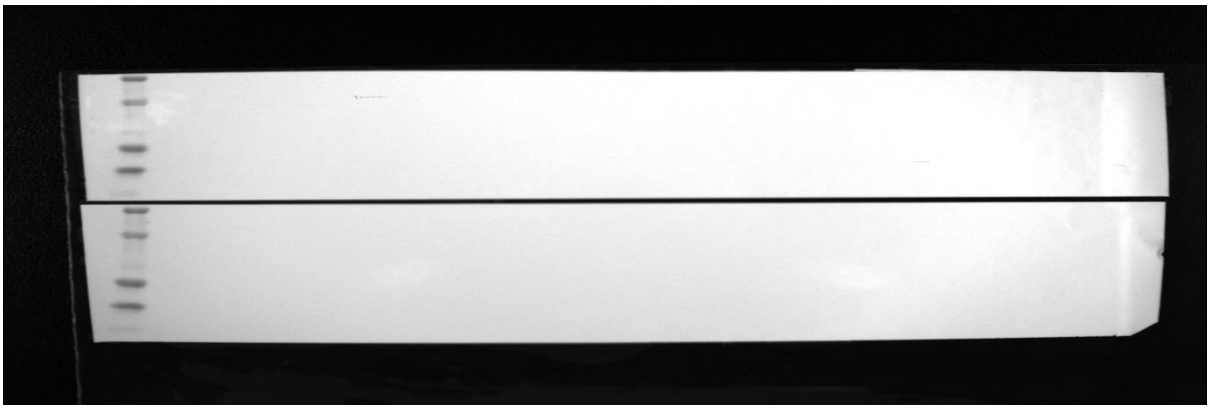

Marker overlay

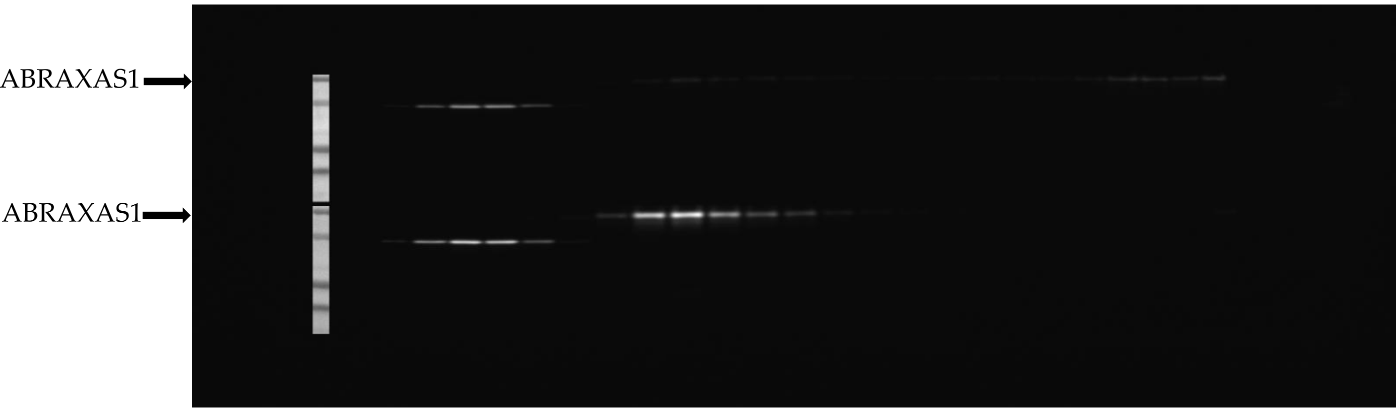

Replicate 2: Top blot: Control gradient; Bottom blot: RNase treated gradient

Molecular weight marker:
- 50 kDa
- 37 kDa
- 25 kDa
- 20 kDa
- 15 kDa
- 10 kDa

Gradient samples

Loading order: protein ladder, Fraction 1,2,3,4…………………………………...………25

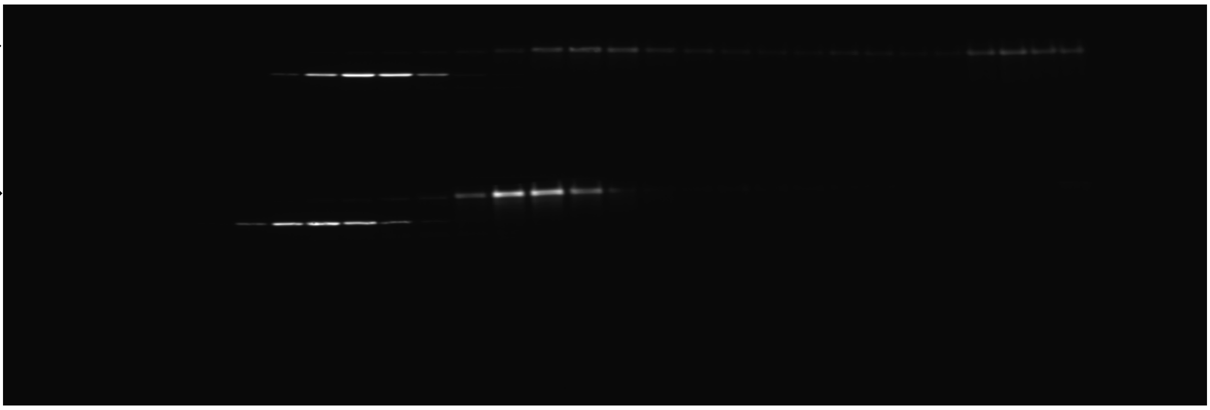

Membrane scan to detect protein ladder

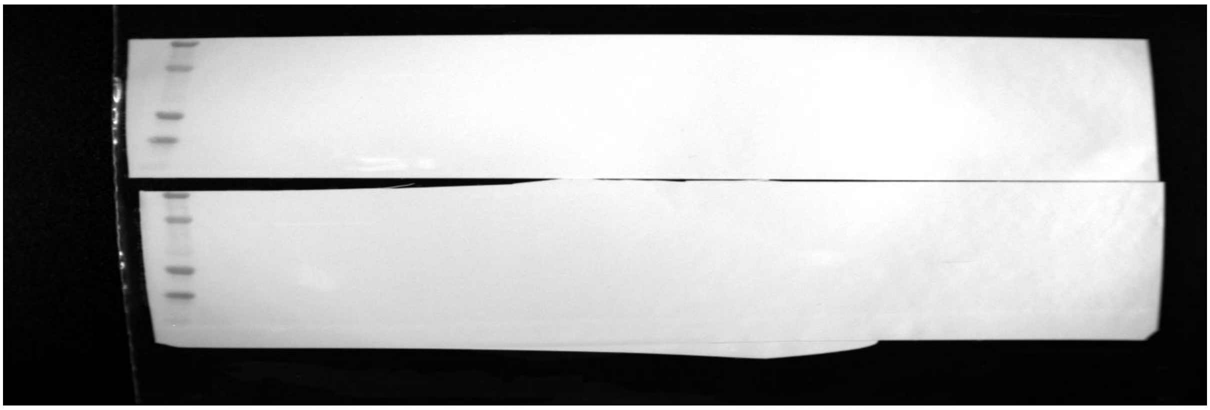

Marker overlay

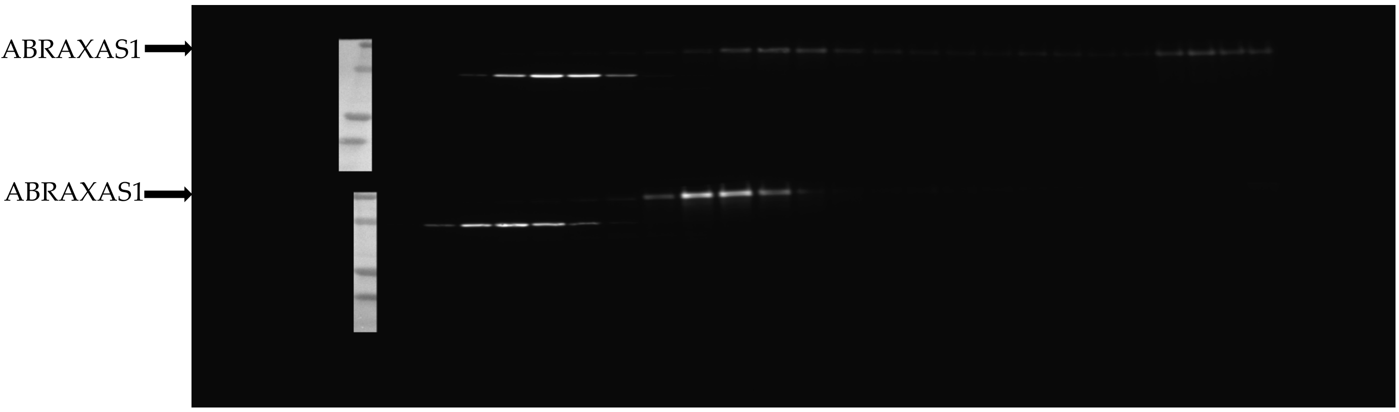

Replicate 3: Top blot: Control gradient; Bottom blot: RNase treated gradient

Molecular weight marker:
50 kDa
37 kDa
25 kDa
20 kDa
15 kDa
10 kDa

Gradient samples

Loading order: protein ladder, Fraction 1,2,3,4………………………………………..………25

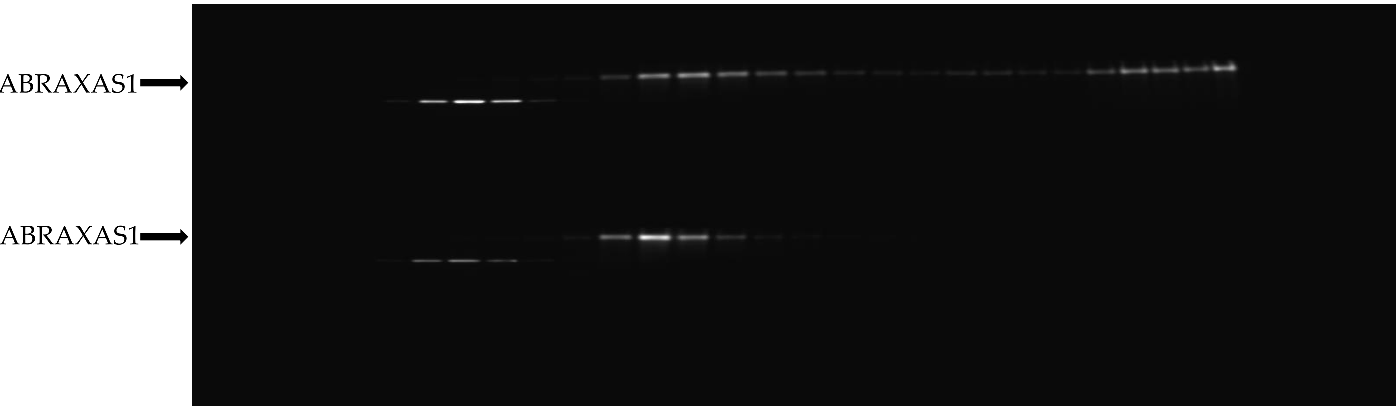

Membrane scan to detect protein ladder

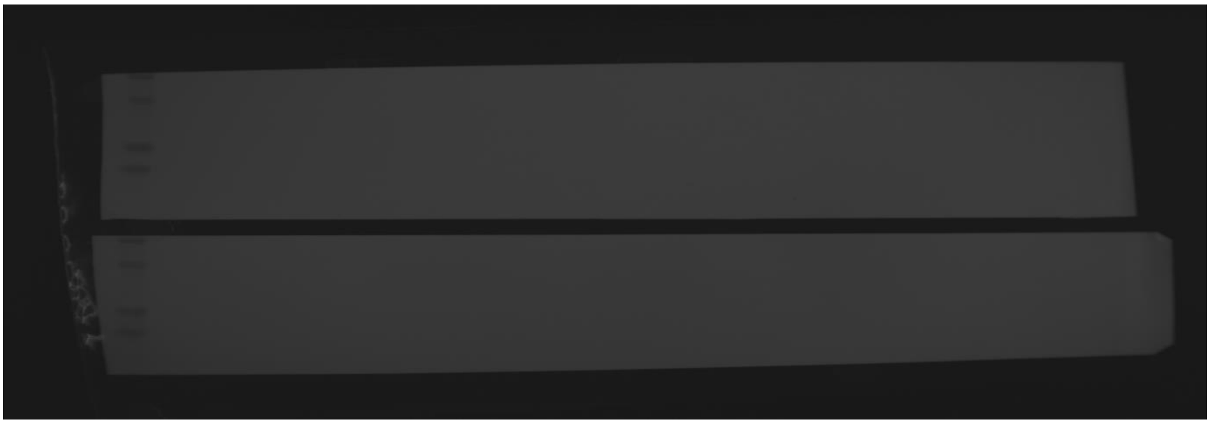

Marker overlay

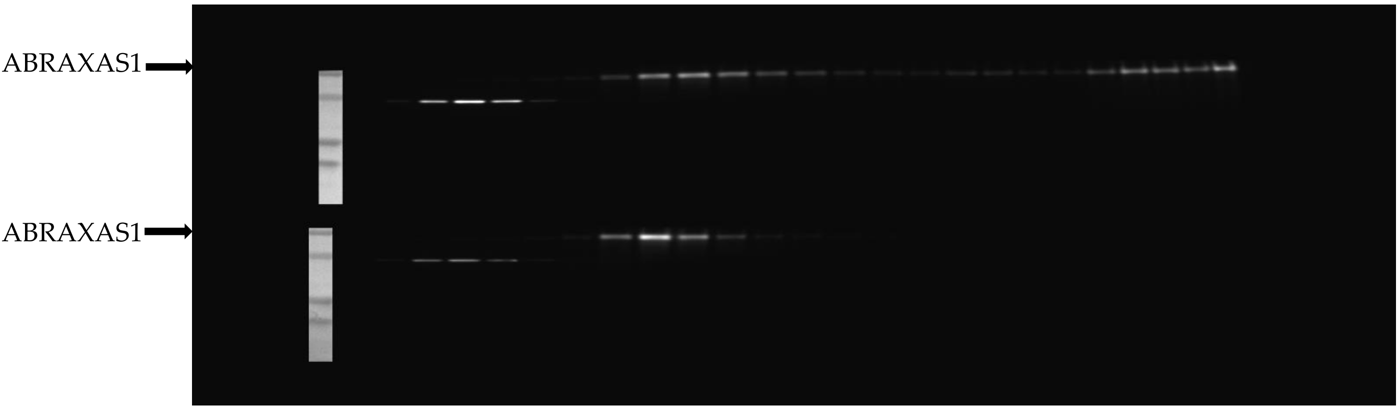

Figure 6D: Direct RNA binding of ABRAXAS1

Autoradiography [32P]

Replicate 1:

UV Crosslinked, 254nm, 200mJ/cm^2 Non-Crosslinked

IgG ABRAXAS1

RNase I dilution 1:5 1:5 1:50 1:500 1:5

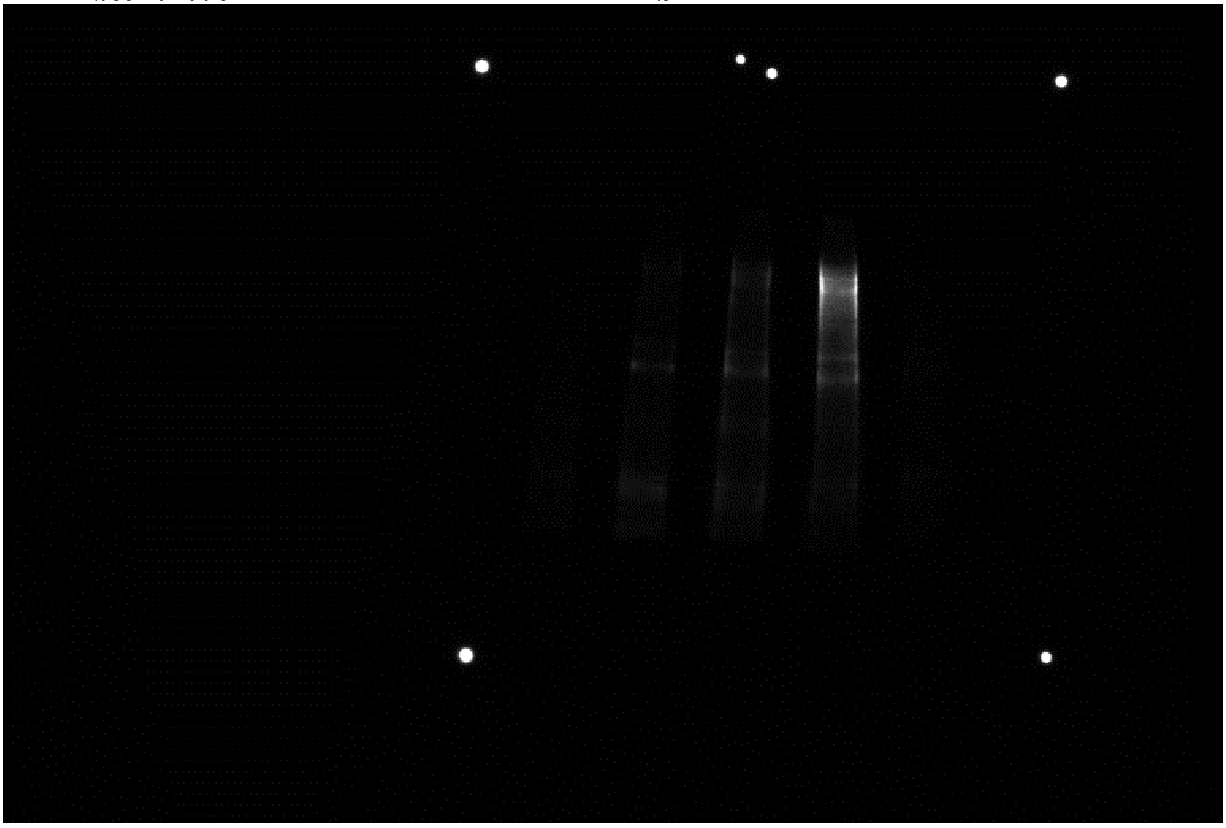

Replicate 2:

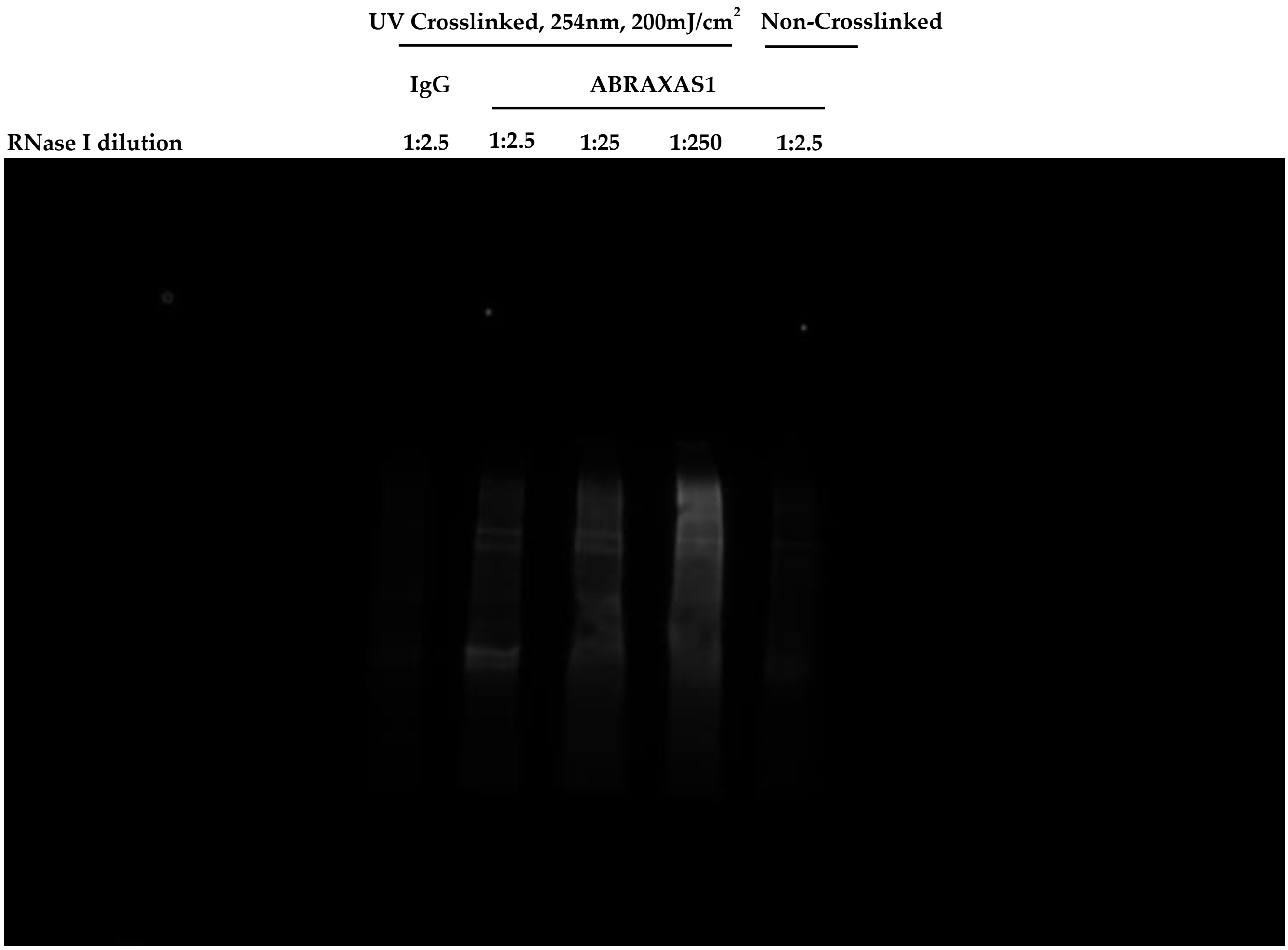
UV Crosslinked, 254nm, 200mJ/cm2
Non-Crosslinked
IgG
ABRAXAS1
RNase I dilution
1:2.5
1:2.5
1:25
1:250
1:2.5

Replicate 3:

UV Crosslinked, 254nm, 200mJ/cm^2 **Non-Crosslinked**

IgG **ABRAXAS1**

RNase I dilution **1:2.5** **1:2.5** **1:25** **1:250** **1:2.5**

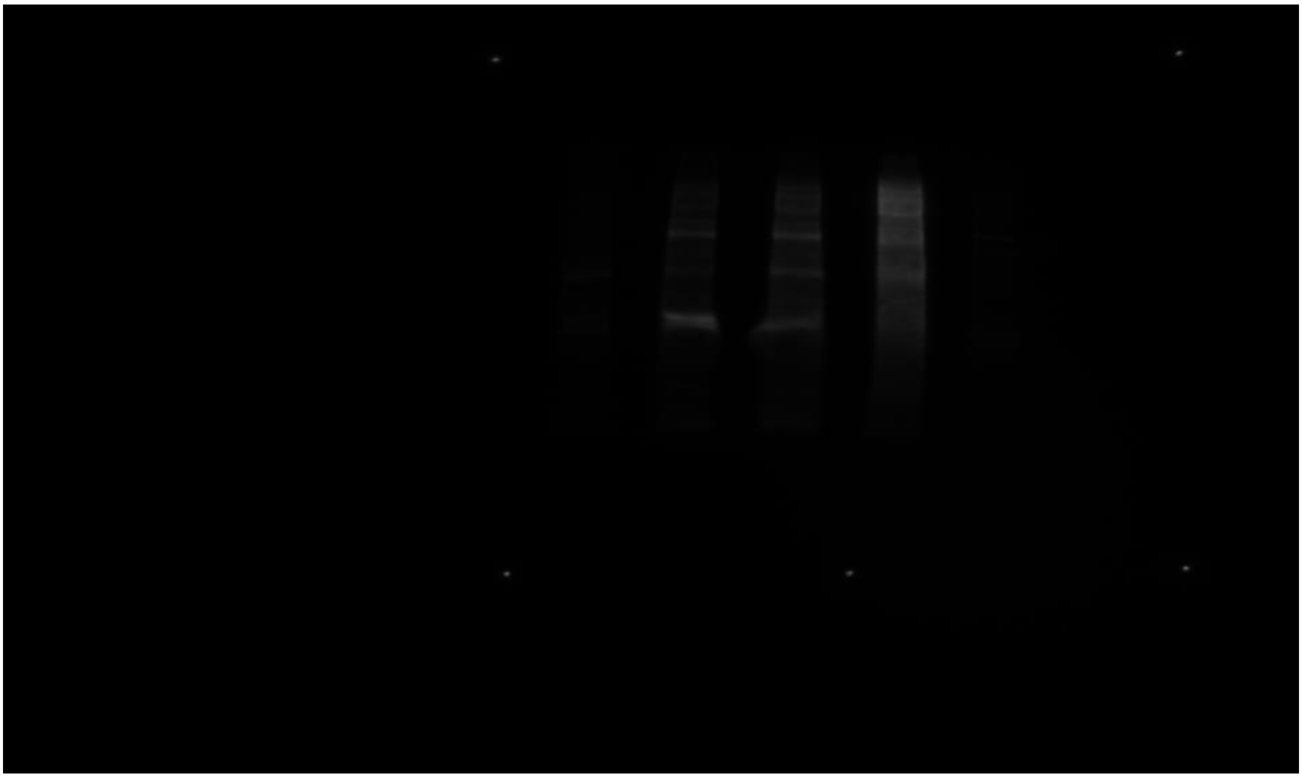

Figure 6E: Immunoprecipitation efficiency of ABRAXAS1 for iCLIP2

Replicate 1:

MW: 47 kDa

Loading order: Marker/Lysate CL, FT IgG 1:5 , IP IgG 1:5, FT ABRAXAS1 1:5, IP ABRAXAS1 1:5, FT ABRAXAS1 1:50, IP ABRAXAS1 1:50, FT ABRAXAS1 1:500, IP ABRAXAS1 1:500, Lysate NCL, FT ABRAXAS1 NCL 1:5, IP ABRAXAS1 NCL 1:5 (Lysate/FT:5%)

Molecular weight marker:
180 kDa
130 kDa
100 kDa
70 kDa
55 kDa
40 kDa
35 kDa
25 kDa
15 kDa
10 kDa

ABRAXAS1
IgG
Heavy chain

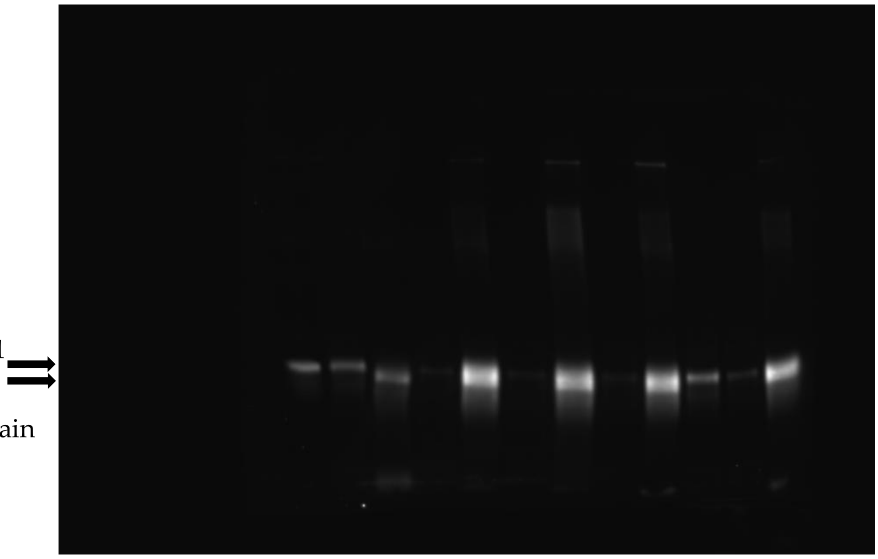

Membrane scan to detect protein ladder

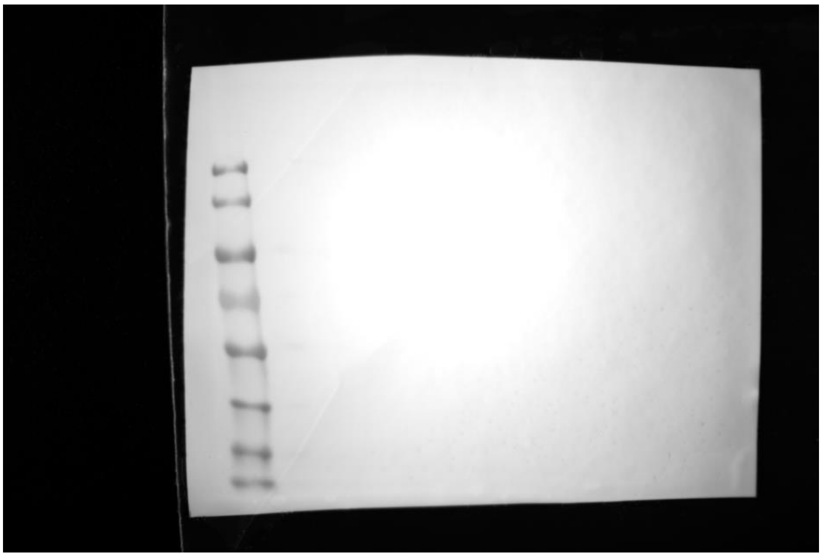

Marker overlay:

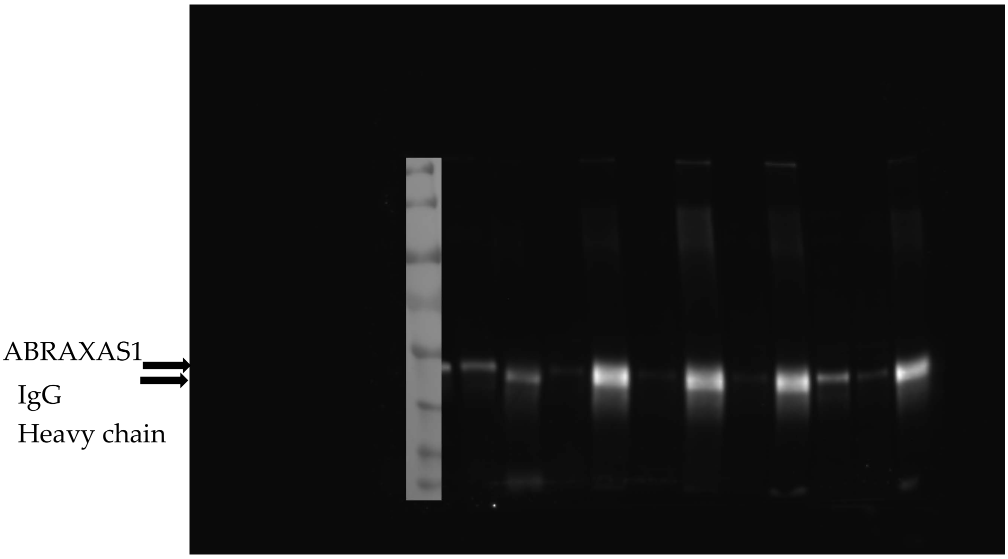

Replicate 2:

MW: 47 kDa

Loading order: Marker/Lysate CL, FT IgG 1:2.5 , IP IgG 1:2.5, FT ABRAXAS1 1:2.5, IP ABRAXAS1 1:2.5, FT ABRAXAS1 1:25, IP ABRAXAS1 1:25, FT ABRAXAS1 1:250, IP ABRAXAS1 1:250, lysate NCL, FT ABRAXAS1 NCL 1:2.5, IP ABRAXAS1 NCL 1:2.5 (Lysate/FT:5%)

Molecular weight marker:
180 kDa
130 kDa
100 kDa
70 kDa
55 kDa
40 kDa
35 kDa
25 kDa
15 kDa
10 kDa

ABRAXAS1
IgG
Heavy chain

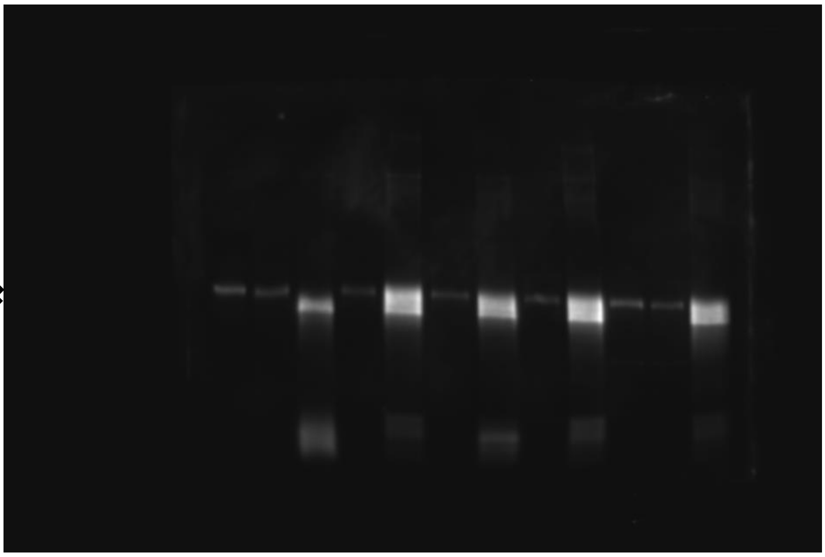

Membrane scan to detect protein ladder

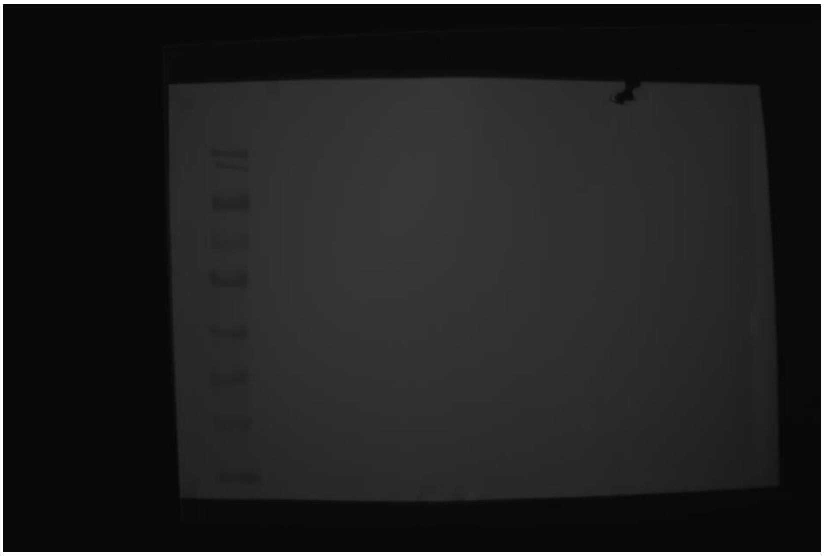

Marker overlay

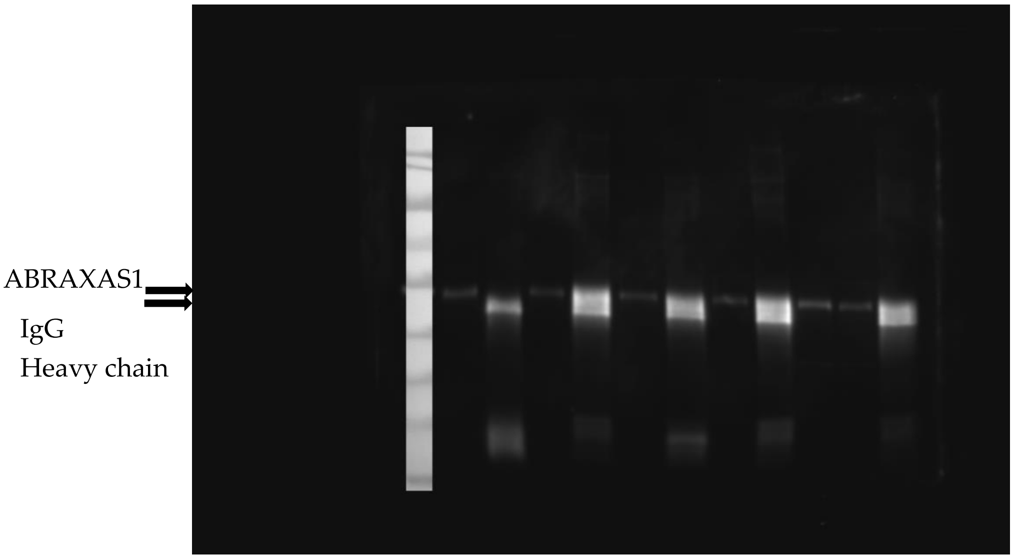

Replicate 3:

MW: 47 kDa

Loading order: Marker/Lysate CL, FT IgG 1:2.5 , IP IgG 1:2.5, FT ABRAXAS1 1:2.5, IP ABRAXAS1 1:2.5, FT ABRAXAS1 1:25, IP ABRAXAS1 1:25, FT ABRAXAS1 1:250, IP ABRAXAS1 1:250, lysate NCL, FT ABRAXAS1 NCL 1:2.5, IP ABRAXAS1 NCL 1:2.5 (Lysate/FT:5%)

Molecular weight marker:
180 kDa
130 kDa
100 kDa
70 kDa
55 kDa
40 kDa
35 kDa
25 kDa
15 kDa
10 kDa

ABRAXAS1
IgG
Heavy chain

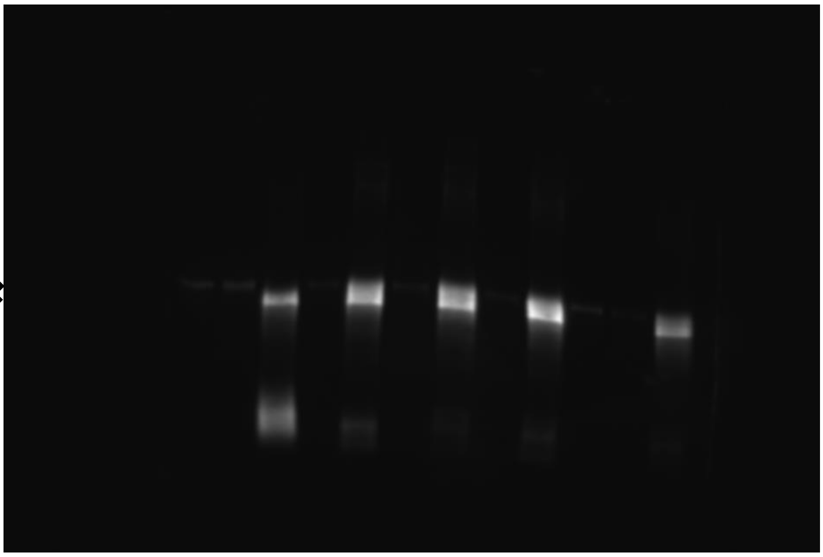

Membrane Scan to detect protein ladder

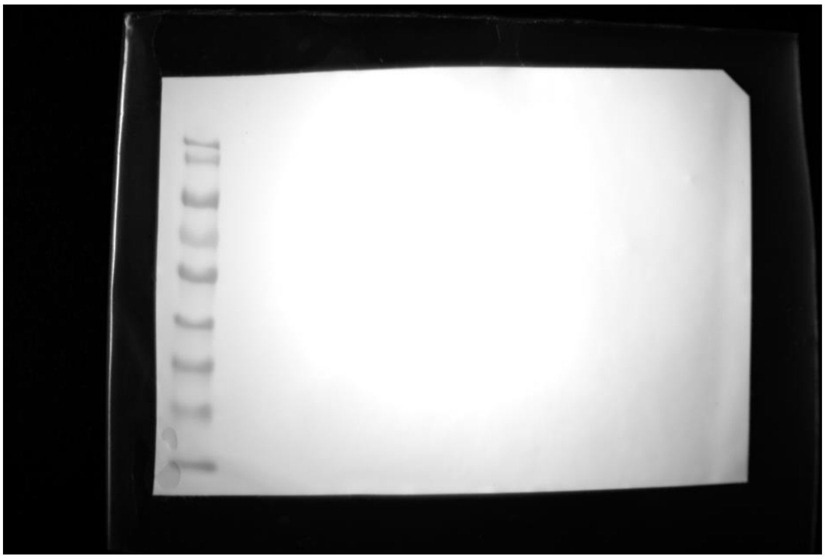

Marker overlay

ABRAXAS1

IgG
Heavy chain

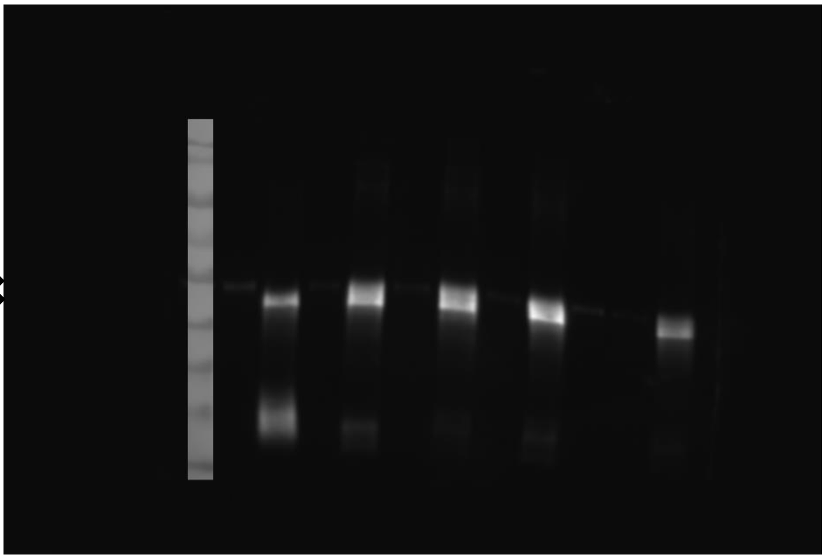

Supplement: Supplementary file 1 [file cancers-14-06109-s001.zip › cancers-2087774-supplementary.pdf]
